# Synergistic association of *STX1A* and *VAMP2* with cryptogenic epilepsy in North Indian population

**DOI:** 10.1002/brb3.490

**Published:** 2016-06-14

**Authors:** Ruchi Baghel, Sandeep Grover, Harpreet Kaur, Ajay Jajodia, Shama Parween, Juhi Sinha, Ankit Srivastava, Achal Kumar Srivastava, Kiran Bala, Puneet Chandna, Suman Kushwaha, Rachna Agarwal, Ritushree Kukreti

**Affiliations:** ^1^ Council of Scientific and Industrial Research (CSIR)Institute of Genomics and Integrative Biology (IGIB)Mall RoadDelhi110 007India; ^2^ Division of Pneumonology‐ImmunologyDepartment of PaediatricsCharité University Medical CentreBerlinGermany; ^3^ Neurology DepartmentNeuroscience CentreAll India Institute of Medical Sciences (AIIMS)New DelhiIndia; ^4^ Institute of Human Behavior & Allied Sciences (IHBAS)Dilshad GardenDelhi110 095India; ^5^ AceProbe Technologies (India) Pvt. Ltd.New DelhiIndia

**Keywords:** Common epilepsy, epistasis, interaction, ion channels, *STX1A*, Synaptic vesicle cycle, *VAMP2*

## Abstract

**Introduction:**

“Common epilepsies”, merely explored for genetics are the most frequent, nonfamilial, sporadic cases in hospitals. Because of their much debated molecular pathology, there is a need to focus on other neuronal pathways including the existing ion channels.

**Methods:**

For this study, a total of 214 epilepsy cases of North Indian ethnicity comprising 59.81% generalized, 40.19% focal seizures, and based on epilepsy types, 17.29% idiopathic, 37.38% cryptogenic, and 45.33% symptomatic were enrolled. Additionally, 170 unrelated healthy individuals were also enrolled. Here, we hypothesize the involvement of epilepsy pathophysiology genes, that is, synaptic vesicle cycle, SVC genes (presynapse), ion channels and their functionally related genes (postsynapse). An interactive analysis was initially performed in SVC genes using multifactor dimensionality reduction (MDR). Further, in order to understand the influence of ion channels and their functionally related genes, their interaction analysis with SVC genes was also performed.

**Results:**

A significant interactive two‐locus model of *STX1A*_rs4363087|*VAMP2*_rs2278637 (presynaptic genes) was observed among SVC variants in all epilepsy cases (*P*_1000_‐value = 0.054; CVC = 9/10; OR = 2.86, 95%CI = 1.88–4.35). Further, subgroup analysis revealed stronger interaction for the same model in cryptogenic epilepsy patients only (*P*_1000_‐value = 0.012; CVC = 10/10; OR = 4.59, 95%CI = 2.57–8.22). However, interactive analysis of presynaptic and postsynaptic genes did not show any significant association.

**Conclusions:**

Significant synergistic interaction of SVC genes revealed the possible functional relatedness of presynapse with pathophysiology of cryptogenic epilepsy. Further, to establish the clinical utility of the results, replication in a large and similar phenotypic group of patients is warranted.

## Introduction

Majority epilepsy patients in the hospitals belong to “common epilepsies” which are complex, sporadic, and nonfamilial in nature (Greenberg and Subaran 2011; Sisodiya and Mefford 2011). However, to date a variety of research has been published on rare Mendelian epilepsies and possibility of application of their findings to the common epilepsies has remained elusive (Johnson and Shorvon 2011). Therefore, genetic dissection of common epilepsies by utilizing advanced molecular and statistical analytical techniques is highly required (Tan et al. 2004). The epilepsy genetics paradigm currently holds epilepsies as “channelopathies” with disturbances observed in both, glutamatergic (excitatory) and GABAergic (inhibitory) neurotransmission. Of all the ion channels, voltage‐gated sodium channel *SCN1A* has been the most clinically relevant epilepsy gene followed by genes encoding calcium channels (e.g., *CACNA1E*). (Schlachter et al. 2009; Parihar and Ganesh 2013). Among the neurotransmitter receptors, the inhibitory GABA receptors are majorly studied (Casillas‐Espinosa et al. 2012). From the GABA system, the aldehyde dehydrogenase (encoded by *ALDH5A1*) involved in metabolism of GABA molecule has been reported in idiopathic generalized epilepsy (IGE) (Lorenz et al. 2006). Additionally, significant association was also reported for neurotransmitter transporter *SLC6A*4 variants in mesial temporal lobe epilepsy (Kauffman et al. 2009). Conclusively, no firm role of ion channels, receptors and transporters could be established in common epilepsies, which urges the need to probe additional neuronal pathway genes. (Greenberg and Subaran 2011; Baghel et al. 2014).

As evident from recent literature, complex synapse biology involving neurotransmitter release can also be explored to understand the epilepsy pathophysiology (Bozzi et al. 2012; Casillas‐Espinosa et al. 2012). For a synaptic transmission, the trigger in terms of action potential (input) needs to be formulated into the neurotransmitter release (output) signals, primarily mediated by synaptic vesicle cycle (SVC) genes. So far, few studies focusing on synapsin, syntaxin, and syndapin genes have explored the possible role of SVC genes in epilepsy genetics (Saitsu et al. 2008; Hamdan et al. 2009; Otsuka et al. 2010; Casillas‐Espinosa et al. 2012). As of now studies lack comprehensive gene set from the SVC pathway and, therefore, a thorough gene list from this principle pathway was prioritized for this study. Additionally, assuming that the presynaptic and postsynaptic neuronal machinery may have a complimentary or additive effect in seizure or epilepsy development, other than SVC, genes from ion channels and their functionally related genes were also prioritized. In this study, genotype‐phenotype correlation of common epilepsies was performed, in which gene‐gene interaction/epistasis among variants of SVC genes was explored. Further, to understand the influence of ion channels and their functionally related genes, a merged analysis of all the gene variants was also performed.

## Material and Methods

### Subjects

A total of 214 epilepsy patients of North Indian ethnicity fulfilling the inclusion and exclusion criteria were enrolled in the study (Grover et al. 2010). Based on the inclusion criteria, patients above 5 years of age, either male or female with at least two unprovoked seizures, undergoing treatment with any of the four first generation antiepileptic drugs (AEDs), that is, Phenytoin (PHT), Carbamazepine (CBZ), Valproate (VPA), Phenobarbitone (PB), or their combinations were included. On the basis of the exclusion criteria, patients with gross neurological deficits such as mental retardation, motor deficits and imaging abnormalities including tumor, multiple neurocysticercosis, tuberculoma, vascular malformation, and atrophic lesions were excluded from the study. Additionally, patients with severe hepatic and renal disorders and pregnant women were also excluded. The patients were enrolled from the outpatient department of Neurology at the Institute of Human Behavior and Allied Sciences (IHBAS), Delhi, India. The phenotypic information of patients was collected on the basis of standard questionnaires of the project which included information about gender, age at seizure onset, type of seizure, medication, baseline seizure frequency, AED prescription, neurological examination, biochemical and hematological profiling, brain imaging, seizure frequency *etc*. For diagnosis and classification of seizures and epilepsy types, guidelines of International League Against Epilepsy (ILAE), 1989 and 1981 were followed (Epilepsy, 1981, 1989). Epilepsy types were classified as idiopathic, cryptogenic, and symptomatic. Seizures were classified as generalized and focal. In addition, a total of 170 ethnically matched unrelated healthy individuals were also randomly recruited from the general population of the same region as the epilepsy patients. None of the controls had history of epilepsy or any neurological disorder. All the patients and healthy individuals gave their informed consent for participation in the study. The study was approved by the institutional and biomedical research ethics committees of both the participating centers.

### Gene and SNP prioritization

A total of 76 Single nucleotide polymorphisms (SNPs) across 7 SVC genes including *SNAP25, STX1A, STXBP1, SYN2, SYT1, VAMP2, and EFHC1* were prioritized for this study (Table S1). Further, a total of 133 SNPs from 12 ion channels and their functionally related genes including *ALDH5A1, CACNA1E, GABRA1, GABRA6, GABRB3, GABRG2, GRIK1, GRIN1, SCN1A, SCN1B, SCN2A*, and *SLC6A11* were also prioritized, while SNP prioritization gene coverage, SNP tagging and location (promoter region, untranslated region, exonic, intronic, splice site) of SNPs within gene were taken into consideration. Further, SNPs were also examined for any in‐silico functional role by means of online prediction tools such as PupaSuite and SNPinfo. Additionally, significantly associated SNPs, based on genotype‐phenotype correlation, were later searched for any biological role through HaploReg tool in terms of effect on chromatin states, alteration of regulatory motifs/binding sites and existence in conserved region.

### Genotyping

Genomic DNA was isolated from peripheral blood cells by using a modified version of salting‐out method (Miller et al. 1988). Of the 76 prioritized SNPs of SVC genes, 67 were left after performing assay designing and initial optimization whereas, of the 133 prioritized SNPs of ion channels and their functionally related genes, 119 SNPs were left. These were then genotyped using iPLEX Gold–Sequenom MassARRAY Genetic Analysis System (Sequenom, Inc., San Diego, CA) using matrix‐assisted laser desorption/ionization time‐of‐flight (MALDI‐TOF) mass spectrometry. Later a random 5% of the samples were re‐genotyped to determine precision by sequencing method using the BigDye Terminator kit (version 3.1; Applied Biosystems, Foster City, CA). Homogeneity of the samples was also verified by typing nine randomly chosen autosomal microsatellite markers (D10S548, D10S196, D10S1653, D11S937, D11S901, D13S218, D13S175, D20S115, and D20S107) unrelated to epilepsy. For testing the stratification, genotype frequencies of unlinked markers were compared between cases and controls using Pearson's *χ* ^2^ test. The *P*‐value of the generated test statistics was observed as the fraction of 10,000 simulated statistics and it exceeded the observed value. For each locus, sum of the test statistics was then computed with number of degrees of freedom (df) and was equal to the sum of the number of df of individual loci. This was performed by STRAT software (Pritchard et al. 2000).

### Statistical analysis

Genotyped SNPs were subjected to SNP level and sample level quality checking (QC) based on minor allele Frequency (MAF) <0.01 and Hardy–Weinberg equilibrium (HWE) at *P*‐value<0.001. A total of 52 (out of 67) SNPs of SVC, and 103 (out of 119) SNPs of ion channels and their functionally related genes, were eligible for further statistical analysis. The genetic association testing for all the SNPs was performed by means of logistic regression (dominant model) to reduce multiple corrections and after observing the genotypic data. *P*‐values were adjusted for covariates, age, and gender. Multiple corrections were applied by means of false discovery rate (FDR) approach. The association effect size for respective analysis was estimated in terms of odds ratio (OR) along with 95% confidence interval (95% CI). The STATA (StataCorp. 2009. *Stata Statistical Software: Release 11*. College Station, TX: StataCorp LP) and PLINK (http://pngu.mgh.harvard.edu/purcell/plink/) software packages were used for all statistical analysis.

To address the issue of epistasis in complex disorders, a nonparametric and genetic model free data mining approach was used. This is based on constructive induction process by which all possible combinations of the polymorphisms is constructed. The 10‐fold cross‐validation is used to assess the accuracy, sensitivity, specificity etc. of the model by means of Bayes classifier. A single best model with maximal testing accuracy and cross‐validation consistency (CVC) is predicted. CVC is a useful approach which limits false positives and assesses the generalizability of the model, and the perfect cross‐validation consistency is considered as 10/10 (Moore et al. 2006). The interaction analysis was performed by using an unbiased multifactor dimensionality reduction (MDR) approach (Moore and Williams 2005). MDR reduces the multidimensional genetic data/attributes/SNP to one dimension (constructive induction) (Hahn et al. 2003). Further, the significance of the model is assessed by 1000‐fold permutation, which corrects for multiple testing and permuted *P*‐value (*P*_1000_‐value) is calculated. The final step is the interpretation of multilocus model of disease susceptibility by means of interaction graphs or dendrograms in terms of synergistic and nonsynergistic interactions (Moore et al. 2006).

Statistical epistasis occurs at the population level and existence of it may or may not warrant the existence of biological epistasis. But presence of biomolecular interactions, based on genes from the best models of MDR, can help in better interpretation of results (statistical epistasis). Although due to existing inherent nonlinearity it is often difficult to detect and characterize statistical epistasis in human studies.

## Results

Of the enrolled 214 patients, 128 patients were observed to have generalized type of seizures, whereas 86 patients experienced focal type of seizures. Detailed seizure subtypes of generalized and focal seizures have been mentioned in the demographic table (Table 1). Based on epilepsy type, the patient pool comprised 17.29% idiopathic, 37.38% cryptogenic, and 45.33% symptomatic epilepsy patients. Additionally, our study also included 170 unrelated healthy individuals (Table 1). Mean age of the enrolled patients was 21.92 ± 9.36 SD, whereas mean age of controls was 26.67 ± 7.88 SD. Majority of the patients (49.76%) had onset age ranging from 6 to 15 years. Further, as the stratification issue was addressed by stratification analysis through STRAT software, the results confirmed the homogeneity of samples with *χ* ^2^ = 76.67, df = 60, *P*‐value = 0.072.

**Table 1:** Demographic characteristics of 214 patients with epilepsy enrolled in the study

Phenotypic characteristics	Cases *n* = 214	Controls (*n* = 170)
Age (years)		
Mean ± SD (*n*)	21.92 ± 9.36 (213)	26.67 ± 7.88 (161)
Body weight (Kg)		
Mean ± SD (*n*)	49.16 ± 13.70 (209)	–
Age at seizure onset (years) [*n* (%)]		
<5	18 (8.53)	–
6–15	105 (49.76)	–
16–25	61 (28.91)	–
Above 25	27 (12.80)	–
Gender [*n* (%)]		
Male	135 (63.08)	84 (49.4)
Female	79 (36.92)	86 (50.6)
Seizure type bin [*n* (%)]		
Generalized	128 (59.81)	–
Focal	86 (40.19)	–
Seizure type [*n* (%)]		
Generalized tonic–clonic seizures (GTCS)	114 (53.27)	–
Simple partial seizures (SPS)	5 (2.34)	–
Simple partial seizures with secondary generalization (SPS sec. gen.)	53 (24.77)	–
Complex partial seizures (CPS)	11 (5.14)	–
Complex partial seizures with secondary generalization (CPS sec.gen.)	17 (7.94)	–
Others	14 (6.54)	–
Epilepsy type [*n* (%)]		
Idiopathic	37 (17.29)	–
Cryptogenic	80 (37.38)	–
Symptomatic	97 (45.33)	–

SD, standard deviation; *n*, number.

The single SNP analysis of all the 155 SNPs of SVC, ion‐channels, and their functionally related genes was performed in all epilepsy and subgroups based on, seizures and epilepsy types. The analysis revealed significant associations for few SNPs (Tables S2 and S3) but, none of them could remain significant after correction.

**Figure 1:**
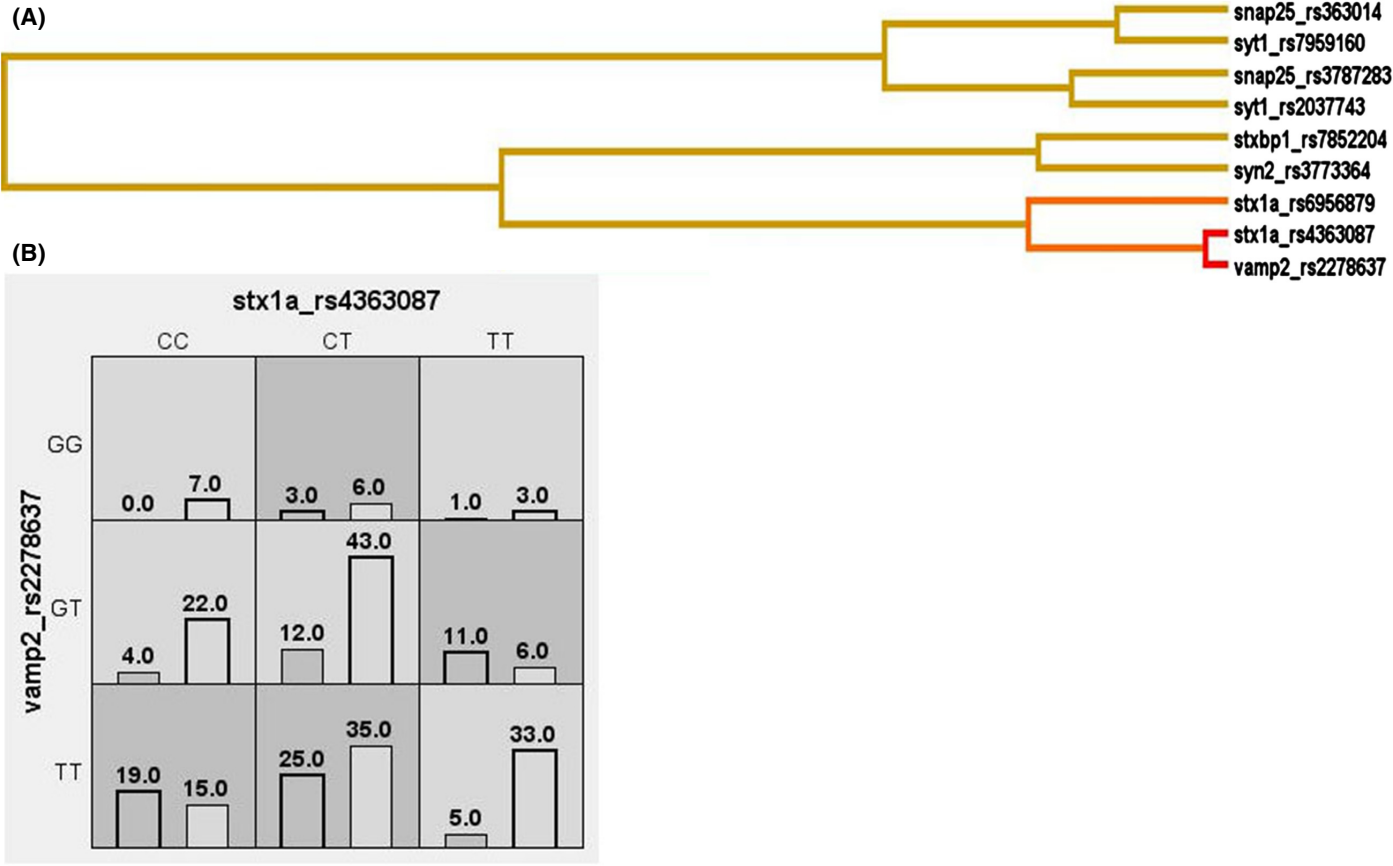
Gene‐gene interaction results for SVC synaptic vesicle cycle genes in cryptogenic epilepsy patients. (A) Depicts the bar graph where high‐risk genotype combinations are in gray and low‐risk genotype combinations are in white. The left bars represent all epilepsy patients and the right bars represent controls. (B) Depicts the dendogram generated from MDR where red or orange line indicates synergistic relationship; golden line represents additivity, and blue or green line represents redundancy. Shorter the length of the dendogram arm, stronger is the interaction.

Further considering the concept of epistasis, interaction analysis was performed by MDR initially in SVC variants. The MDR analysis revealed a significant two‐locus model *STX1A*_rs4363087‐*VAMP2*_rs2278637 with very high CVC value of 9/10 in all epilepsy (OR = 2.86, 95% CI = 1.88–4.35; *P*_1000perm_ = 0.054) (Table 2). On subgroup analysis by seizure subtypes, the same model was also observed in both generalized (CVC = 6/10, OR = 2.62, 95% CI = 1.63–4.20; *P* < 0.0001) and focal (CVC = 9/10, OR = 3.42, 95% CI = 1.98–5.91; *P* < 0.0001) subgroups (Table 2) but none could remain significant after permutation (generalized *P*_1000perm_ = 0.470 and focal *P*_1000perm_ = 0.139). Further, the subgroup analysis by epilepsy subtypes also identified the same two‐locus model with high CVC in cryptogenic epilepsy only (CVC = 10/10, OR = 4.59, 95% CI = 2.57–8.22; *P* < 0.0001) and which also remained significant after permutation *P*_1000perm_ = 0.012 (Table 2, Fig. 1). The subgroup analysis, thus, helped in identifying the true genetic association with high significant permutated *P*‐value in cryptogenic epilepsy. This has highlighted the involvement of unexplored presynaptic gene system in epilepsy pathophysiology.

**Table 2:** Gene‐gene interaction results for best models among *SVC* genes (presynaptic) in all epilepsy patients and different subgroups

Model	Accuracy	Sensitivity	Specificity	Cross‐validation consistency (CVC)*	OR (95% CI)	*P*‐value	*P*‐value 1000 permutation
All Epilepsy patients							
*STX1A*_rs4363087,*VAMP2*_rs2278637	62.24	58.41	67.06	**9/10**	2.86 (1.88–4.35)	*P* < 0.0001	**0.054#**
Focal							
*STX1A*_rs4363087,*VAMP2*_rs2278637	64.45	66.28	63.53	**9/10**	3.42 (1.98–5.91)	*P* < 0.0001	0.139
Cryptogenic							
*STX1A*_rs4363087,*VAMP2*_rs2278637	66.4	72.5	63.53	**10/10**	4.59 (2.57–8.22)	*P* < 0.0001	**0.012#**

All the interaction models were output of multifactor dimensionality reduction (MDR).

*CVC > 8/10, # *P*‐values remained significant after permutation.

Individually, ion channels and their functionally related genes were also independently reviewed for interaction analysis in all epilepsy and subgroups. A two‐locus model *GABRB3*_rs878960‐*SCN1A*_rs1813502 with high CVC = 10/10 (OR = 3.27, 95% CI = 2.01–5.30; *P* < 0.0001) with generalized seizure patients was observed, but could not remain significant after 1000 permutation *P*_1000perm_ = 0.490 (Table S4). No other model had high CVC value.

**Figure 2:**
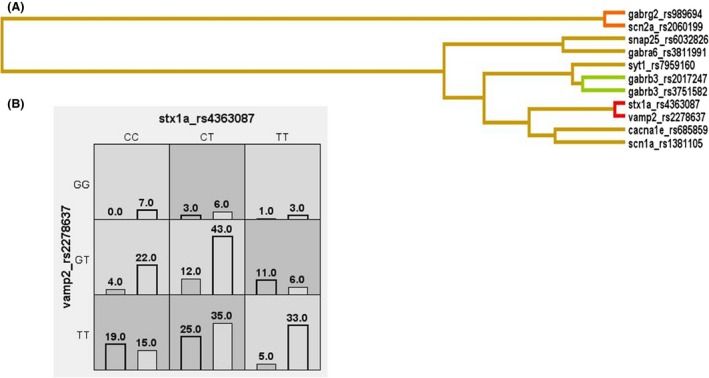
Gene‐gene interaction results for merged gene set of SVC and ion channels and their functionally related genes in cryptogenic epilepsy patients. (A) Depicts the bar graph where high‐risk genotype combinations are in gray and low‐risk genotype combinations are in white. The left bars represent all epilepsy patients and the right bars represent controls. (B) Depicts the dendogram generated from MDR where red or orange line indicates synergistic relationship; golden line represents additivity, and blue or green line represents redundancy. Shorter the length of the dendogram arm, stronger is the interaction.

In order to better understand the brain mechanisms behind epilepsy and seizure generation, the ion channels and their functionally related gene variants were merged with SVC variants for interaction analysis. Any significant model comprising both the gene sets could hint toward the possible biological phenomenon involving both presynaptic and postsynaptic neuronal processes. The same two‐locus model of *GABRB3*_rs878960‐*SCN1A*_rs1813502 of ion channels and their functionally related genes was, however, also observed in generalized seizure patients but with low CVC value of 6/10. Additionally, the same two‐locus model of *STX1A*_rs4363087‐*VAMP2*_rs2278637 of presynapse genes was observed to be significant in all epilepsy patients (CVC = 7/10, OR = 2.86, 95% CI = 1.88–4.35; *P* < 0.0001) but it did not remaine significant after permutation *P*_1000perm_ = 0.342. Further subgroup analysis in seizures and epilepsy type also revealed the significance of the same two‐locus model of *STX1A*_rs4363087‐*VAMP2*_rs227863 of presynapse genes in cryptogenic epilepsy only with CVC = 10/10, OR = 4.59, 95% CI = 2.57–8.22; *P* < 0.0001 which remained significant after permutation *P*_1000perm_ = 0.019 (Table 3, Fig. 2). However, no interactive association of presynapse and postsynapse could be observed; but the results highlighted the independent synergistic effect of presynaptic genes on epilepsy pathophysiology.

**Table 3:** Gene‐gene interaction results for best model among presynaptic and postsynaptic genes in all epilepsy patients and different subgroups

Model	Accuracy	Sensitivity	Specificity	Cross‐validation consistency (CVC)*	OR (95% CI)	*P*‐value	*P*‐value 1000 permutation
Cryptogenic							
*STX1A*_rs4363087,*VAMP2*_rs2278637	66.4	72.5	63.53	**10/10**	4.59 (2.57–8.22)	*P* < 0.0001	**0.019#**

The interaction model was an output of multifactor dimensionality reduction (MDR).

*CVC > 8/10, # *P*‐values remained significant after permutation

## Discussion

The aim of this study was to evaluate the interactive role of SVC, ion channels and their functionally related gene variants in sporadic common forms of epilepsy. The principal finding of this study was the two‐locus model of *VAMP2* and *STX1A* SNPs highlighting synergistic effect of two presynaptic genes on cryptogenic epilepsies. To the best of our knowledge, our study represents the first report of interactive effect of presynaptic genes on epilepsy pathophysiology.

These two genes belong to the soluble NSF (N‐Ethylmaleimide‐sensitive factor) attachment protein receptor (SNARE) complex of SVC pathway; thus, having a role in vesicle priming, fusion and release of neurotransmitters. Polymorphisms from SNARE complex genes have been earlier studied in various neuropsychiatric disorders. According to the reports of study performed on schizophrenia patients, results did not reveal any association with *VAMP2* or *STX1A* (Kawashima et al. 2008). *VAMP2* also did not reveal any significant role of genetics in the etiology of bipolar disorder; however, no distinct relation could be observed (Abou Jamra et al. 2008). For *STX1A,* literature reports have highlighted a significant association of rs4363087 with migraine (Tropeano et al. 2012). With respect to functionality, *VAMP2* is essential for fast Ca^2+^ triggered release, because *VAMP2* knock‐out mice lacks fast Ca^2+^ triggered neurotransmitter release, whereas, slow release phase persists in them (Schoch et al. 2001). Also, *VAMP2* is essential for a large component of the readily releasable pool; hence, it is the key component of the regulated secretory pathway of presynaptic neurons.

Other than neuropsychiatric disorders, role of presynaptic genes have also been reported in epilepsy genetics. Recently predisposing effects of *SYN1* gene have been reported in partial epilepsy (Fassio et al. 2011). Another study by Cavalleri et al. has reported the association of rs3773364 of *SYN2* gene with patients having history of febrile seizures in Caucasian cohorts (Casillas‐Espinosa et al. 2012). The same variant of *SYN2* was also reported as conferring risk to epilepsy in North Indian cohort (Casillas‐Espinosa et al. 2012). Saitsu et al. (2008) have demonstrated association of *STXBP1* variant in Ohtahara syndrome (OS), which was followed by another report of association by Hamdan et al. (2009) in patients with nonsyndromic complex partial epilepsy. Otsuka et al. (2010) reported significant results of *STXBP1* not only in pathogenesis of OS but also in the pathogenesis of West syndrome. Recently Milh et al. (2011) have also reported the possible influence of *STXBP1* gene in early onset epileptic encephalopathy. For syndapin 1, an indirect link was found, as it is present on the locus 6p21.3, which is associated with genetic generalized epilepsies (Casillas‐Espinosa et al. 2012). Yilmaz et al. (2014) have recently reported the association of insertion/deletion of *VAMP2* and 33 bp promoter region of synaptotagmin‐1 with IGE patients. Additionally, mouse model reports have also linked dynamins and synaptosomal‐associated proteins of SVC with missense mutation of dynamin (Casillas‐Espinosa et al. 2012). Another gene *SNAP‐25,* present on autosomal deletion region of chromosome 2 in neurological mouse mutant, is known to exhibit typical electrophysiological features of absence epilepsy (Casillas‐Espinosa et al. 2012; Baghel et al. 2014).

Until the availability of HaploReg data, it was majorly unknown that how the noncoding variants could influence the molecular biology of the gene, leading to the development of a diseased trait (Ward and Kellis 2012). Based on haploRegv2, the intronic SNP of *STX1A* has been annotated at histone enhancer marks identified in H1 and K562 cell line which can lead to increased gene expression of *STX1A*. Allele specific differential expression of genes, owing to enhancer elements has been reported in literature (Visser et al. 2012). However, the significance of the current results should not remain limited to the SNPs from the respective genes, rather the interaction of *STX1A* and *VAMP2* should also be kept in consideration while performing replication or further studies. As associated SNP could only be a marker rather than the actual causative variant, a detailed study of several genes may lead to identification of the actual causal variant as well as possible epistatic effect.

This study lays the foundation for future research involving interactive effect of presynaptic genes, thus providing better understanding of genetic etiology of epilepsy. Although we used a powerful and unbiased approach to explain the apparent subgroup effect seen in our study, our results need to be consistently replicated in additional studies in other ethnically similar as well as dissimilar populations (Sun et al. 2014). One of the limitation of our study could be that our priori hypotheses were not in a specific direction and may not be able to support any pre‐existing biological support for differential effect in different subgroups. Lastly, stringent inclusion and exclusion criteria adopted in this study resulted in small patient size. It is quite possible that modest interactive associations observed in the cryptogenic subgroup are perhaps false positive. This further necessitates replication studies in larger cohorts. Additional experimental and animal model studies may be required to understand the underlying functional basis of our subgroup‐specific association. Furthermore, functional validation is warranted for establishing clinical significance of the reported interaction.

## Conflict of Interest

None declared.

## Supporting information


**Table S1.** Gene‐wise summarized status of SNPs for final genotype‐phenotype correlation.
**Table S2.** Association of variants in all epilepsy patients and seizure type subgroups.
**Table S3.** Association of variants in all epilepsy patients and epilepsy type subgroups.
**Table S4.** Gene‐gene interaction results for best models among SVC and ion channel genes in all epilepsy patients and different subgroups.
