# Supplementary material for: Synergistic association of STX1A and VAMP2 with cryptogenic epilepsy in North Indian population
Source: Brain Behav. 2016 Jun 14;6(7):e00490. doi: 10.1002/brb3.490 (PMC4951625; doi:10.1002/brb3.490)
Supplement: Supplementary file 1 — Table S1. Gene‐wise summarized status of SNPs for final genotype‐phenotype correlation. Table S2. Association of variants in all epilepsy patients and seizure type subgroups. Table S3. Association of variants in all epilepsy patients and epilepsy type subgroups. Table S4. Gene‐gene interaction results for best models among SVC and ion channel genes in all epilepsy patients and different subgroups. [file BRB3-6-e00490-s001.docx]

| **Table S1. Gene wise summarized status of SNPs for final genotype phenotype correlation** | | | | | | | | | | |
| --- | --- | --- | --- | --- | --- | --- | --- | --- | --- | --- |
| **Category** | **Gene** | **Gene full name** | **Chromosome No.** | **Total prioritized SNPs** | **Total passed while assay designing** | **Total passed while reaction optimization** | **Total failed** | **No. of non polymorphic (MAF < 0.01)** | **No. of SNPs failed HWE (P < 0.001)** | **Final for analysis** |
| **Ion channels** | **ALDH5A1** | Aldehydedehydrogese 5 family,Membera1 | 6 | 12 | 12 | 11 | 0 | 1 | 0 | 10 |
|  | **CACNA1E** | Calcium channel, voltage-dependent, R type, alpha 1E subunit | 1 | 10 | 10 | 8 | 0 | 1 | 0 | 7 |
|  | **GABRA1** | Gamma-aminobutyric acid (GABA) A receptor, alpha 1 | 5 | 12 | 12 | 12 | 0 | 0 | 0 | 12 |
|  | **GABRA6** | GABA Subunit A Receptor alpha 6 | 5 | 10 | 10 | 10 | 1 | 0 | 1 | 8 |
|  | **GABRB3** | Gamma-aminobutyricacid (GABA) A Receptor,beta 3 | 15 | 11 | 10 | 10 | 0 | 1 | 0 | 9 |
|  | **GABRG2** | Gamma-aminobutyricacid (GABA) A Receptor,gamma 2 | 5 | 15 | 14 | 14 | 2 | 0 | 0 | 12 |
|  | **GRIK1** | Glutamate Receptor,ionotropic, kaite 1 | 21 | 12 | 11 | 9 | 0 | 1 | 1 | 7 |
|  | **GRIN1** | GlutamateReceptor,ionotropic, N-methyld-aspartate 1 | 9 | 10 | 10 | 8 | 0 | 1 | 0 | 7 |
|  | **SCN1A** | Sodium Channel, voltage-gated, type1,alpha Subunit | 2 | 15 | 14 | 14 | 2 | 1 | 0 | 11 |
|  | **SCN1B** | Sodium Channel, voltage-gated, type1,beta subunit | 19 | 11 | 8 | 8 | 0 | 2 | 0 | 6 |
|  | **SCN2A** | Sodium Channel, voltage-gated, type2,alpha Subunit | 2 | 15 | 14 | 14 | 0 | 1 | 0 | 13 |
|  | **SLC6A11** | Solute carrier family 6 (neurotransmitter transporter, GABA), member 11 | 3 | 1 | 1 | 1 | 0 | 0 | 0 | 1 |
|  |  |  |  | **134** | **126** | **119** | **5** | **9** | **2** | **103** |
| **Synaptic vesicle cycle** | **SNAP25** | Synaptosomal-associated protein | 20 | 13 | 11 | 11 | 0 | 0 | 0 | 11 |
|  | **STX1A** | Syntaxin 1A (brain) | 7 | 9 | 8 | 7 | 1 | 0 | 0 | 6 |
|  | **STXBP1** | Syntaxinbinding Protein 1 | 9 | 11 | 11 | 11 | 0 | 3 | 0 | 8 |
|  | **SYN2** | Synapsin 2 | 3 | 13 | 13 | 11 | 0 | 3 | 0 | 8 |
|  | **SYT1** | Synaptotagmin 1 | 12 | 8 | 8 | 8 | 1 | 1 | 0 | 6 |
|  | **VAMP2** | Vesicle-associated membrane protein 2 (synaptobrevin 2) | 17 | 8 | 8 | 5 | 0 | 1 | 0 | 4 |
|  | **EFHC1** | EF-Hand Domain (C-Terminal) Containing 1 | 6 | 14 | 14 | 14 | 0 | 5 | 0 | 9 |
|  |  |  |  | **76** | **73** | **67** | **2** | **13** | **0** | **52** |
| **Total** |  |  |  | **210** | **199** | **186** | **7** | **22** | **2** | **155** |

**MAF: Minor Allele Frequency; HWE: Hardy Weinberg Equilibrium**

| **Table S2. Association of variants in all epilepsy patients and seizure type subgroups** | | | | | | | | | | | | | | | | | |
| --- | --- | --- | --- | --- | --- | --- | --- | --- | --- | --- | --- | --- | --- | --- | --- | --- | --- |
|  |  |  | **All epilepsy cases Vs Controls** | | | | | **Generalized seizures Vs Controls** | | | | | **Focal seizures Vs Controls** | | | | |
| **Gene Name** | **SNP id** | **Dominant model** | **case** | **control** | **OR** | **p-value** | **FDR P-value** | **case** | **control** | **OR** | **p-value** | **FDR P-value** | **case** | **control** | **OR** | **p-value** | **FDR P-value** |
| ***CACNA1E*** | **rs34488539** | **CC/ TC+TT** | 129/84 (60.60/39.40) | 102/59 (63.40/36.60) | 0.82 (0.53-1.28) | 0.381 | 0.846 | 73/54 (57.50/42.50) | 102/59 (63.40/36.60) | 0.74 (0.45-1.21) | 0.226 | 0.858 | 56/30 (65.10/34.90) | 102/59 (63.40/36.60) | 1.13 (0.63-2.03) | 0.692 | 0.900 |
|  | **rs4652678** | **TT/ TC+CC** | 88/125 (41.30/58.70) | 72/89 (44.70/55.30) | 0.79 (0.51-1.22) | 0.287 | 0.798 | 46/81 (36.20/63.80) | 72/89 (44.70/55.30) | 0.64 (0.38-1.05) | 0.074 | 0.743 | 42/44 (48.80/51.20) | 72/89 (44.70/55.30) | 1.28 (0.72-2.26) | 0.395 | 0.815 |
|  | **rs199930** | **CC/ TC+TT** | 94/119 (44.10/55.90) | 76/85 (47.20/52.80) | 0.86 (0.56-1.32) | 0.491 | 0.864 | 49/78 (38.60/61.40) | 76/85 (47.20/52.80) | 0.67 (0.41-1.10) | 0.113 | 0.747 | 45/41 (52.30/47.70) | 76/85 (47.20/52.80) | 1.47 (0.83-2.61) | 0.182 | 0.645 |
|  | **rs704326** | **CC/ TC+TT** | 130/83 (61.00/39.00) | 89/72 (55.30/44.70) | 1.42 (0.91-2.2) | 0.122 | 0.734 | 83/44 (65.40/34.60) | 89/72 (55.30/44.70) | 1.68 (1.02-2.77) | 0.042 | 0.604 | 47/39 (54.70/45.30) | 89/72 (55.30/44.70) | 1.01 (0.57-1.81) | 0.96 | 0.976 |
|  | **rs2280869** | **TT/ TC+CC** | 159/54 (74.60/25.40) | 120/41 (74.50/25.50) | 0.91 (0.56-1.5) | 0.72 | 0.929 | 90/37 (70.90/29.10) | 120/41 (74.50/25.50) | 0.76 (0.44-1.31) | 0.324 | 0.862 | 69/17 (80.20/19.80) | 120/41 (74.50/25.50) | 1.28 (0.65-2.55) | 0.475 | 0.815 |
|  | **rs590412** | **CC/AC+AA** | 80/133 (37.60/62.40) | 52/109 (32.30/67.70) | 1.24 (0.79-1.96) | 0.346 | 0.843 | 45/82 (35.40/64.60) | 52/109 (32.30/67.70) | 1.18 (0.70-1.96) | 0.536 | 0.866 | 35/51 (40.70/59.30) | 52/109 (32.30/67.70) | 1.38 (0.77-2.49) | 0.281 | 0.721 |
|  | **rs685859** | **GG/ GC+CC** | 75/138 (35.20/64.80) | 44/117 (27.30/72.70) | 1.36 (0.86-2.18) | 0.19 | 0.782 | 43/84 (33.90/66.10) | 44/117 (27.30/72.70) | 1.32 (0.78-2.22) | 0.306 | 0.862 | 32/54 (37.20/62.80) | 44/117 (27.30/72.70) | 1.47 (0.80-2.70) | 0.212 | 0.645 |
| ***SCN2A*** | **rs6755708** | **TT/TC+CC** | 139/74 (65.30/34.70) | 87/74 (54.00/46.00) | 1.52 (0.98-2.37) | 0.06 | 0.734 | 81/46 (63.80/36.20) | 87/74 (54.00/46.00) | 1.44 (0.88-2.36) | 0.149 | 0.823 | 58/28 (67.40/32.60) | 87/74 (54.00/46.00) | 1.79 (0.99-3.23) | 0.05 | 0.502 |
|  | **rs6718960** | **AA/AG+GG** | 159/54 (74.60/25.40) | 121/40 (75.20/24.80) | 0.98 (0.6-1.62) | 0.948 | 0.986 | 94/33 (74.00/26.00) | 121/40 (75.20/24.80) | 0.96 (0.55-1.68) | 0.894 | 0.951 | 65/21 (75.60/24.40) | 121/40 (75.20/24.80) | 1.02 (0.53-1.96) | 0.96 | 0.976 |
|  | **rs13432006** | **AA/AG+GG** | 98/115 (46.00/54.00) | 79/82 (49.10/50.90) | 0.9 (0.58-1.38) | 0.627 | 0.919 | 64/63 (50.40/49.60) | 79/82 (49.10/50.90) | 1.05 (0.65-1.71) | 0.843 | 0.945 | 34/52 (39.50/60.50) | 79/82 (49.10/50.90) | 0.66 (0.37-1.18) | 0.163 | 0.645 |
|  | **rs353119** | **GG/AG+AA** | 105/108 (49.30/50.70) | 81/80 (50.30/49.70) | 0.95 (0.62-1.46) | 0.818 | 0.929 | 56/71 (44.10/55.90) | 81/80 (50.30/49.70) | 0.76 (0.47-1.24) | 0.270 | 0.862 | 49/37 (57.00/43.00) | 81/80 (50.30/49.70) | 1.39 (0.79-2.47) | 0.252 | 0.694 |
|  | **rs16850331** | **CC/TC+TT** | 120/93 (56.30/43.70) | 98/63 (60.90/39.10) | 0.73 (0.47-1.13) | 0.154 | 0.752 | 80/47 (63.00/37.00) | 98/63 (60.90/39.10) | 0.97 (0.58-1.60) | 0.893 | 0.951 | 40/46 (46.50/53.50) | 98/63 (60.90/39.10) | 0.48 (0.27-0.86) | 0.013 | 0.502 |
|  | **rs2075703** | **AA/AG+GG** | 115/98 (54.00/46.00) | 91/70 (56.50/43.50) | 0.83 (0.54-1.28) | 0.401 | 0.864 | 77/50 (60.60/39.40) | 91/70 (56.50/43.50) | 1.09 (0.67-1.78) | 0.733 | 0.902 | 38/48 (44.20/55.80) | 91/70 (56.50/43.50) | 0.56 (0.31-0.98) | 0.043 | 0.502 |
|  | **rs2075704** | **GG/AG+AA** | 110/103 (51.60/48.40) | 79/82 (49.10/50.90) | 1.15 (0.75-1.76) | 0.533 | 0.869 | 62/65 (48.80/51.20) | 79/82 (49.10/50.90) | 1 (0.61-1.62) | 0.991 | 0.998 | 48/38 (55.80/44.20) | 79/82 (49.10/50.90) | 1.45 (0.82-2.56) | 0.204 | 0.645 |
|  | **rs1947114** | **TT/TC+CC** | 118/95 (55.40/44.60) | 99/62 (61.50/38.50) | 0.69 (0.45-1.08) | 0.103 | 0.734 | 78/49 (61.40/38.60) | 99/62 (61.50/38.50) | 0.89 (0.54-1.47) | 0.655 | 0.873 | 40/46 (46.50/53.50) | 99/62 (61.50/38.50) | 0.48 (0.27-0.86) | 0.012 | 0.502 |
|  | **rs935403** | **AA/AG+GG** | 80/133 (37.60/62.40) | 65/96 (40.40/59.60) | 0.94 (0.6-1.46) | 0.776 | 0.929 | 44/83 (34.60/65.40) | 65/96 (40.40/59.60) | 0.8 (0.48-1.32) | 0.381 | 0.862 | 36/50 (41.90/58.10) | 65/96 (40.40/59.60) | 1.23 (0.69-2.19) | 0.49 | 0.815 |
|  | **rs2060199** | **AA/AT+TT** | 48/165 (22.50/77.50) | 41/120 (25.50/74.50) | 0.97 (0.58-1.62) | 0.911 | 0.964 | 26/101 (20.50/79.50) | 41/120 (25.50/74.50) | 0.82 (0.46-1.48) | 0.510 | 0.862 | 22/64 (25.60/74.40) | 41/120 (25.50/74.50) | 1.33 (0.68-2.57) | 0.407 | 0.815 |
|  | **rs3943809** | **AA/AG+GG** | 130/83 (61.00/39.00) | 103/58 (64.00/36.00) | 0.74 (0.47-1.17) | 0.196 | 0.782 | 84/43 (66.10/33.90) | 103/58 (64.00/36.00) | 0.95 (0.57-1.59) | 0.845 | 0.945 | 46/40 (53.50/46.50) | 103/58 (64.00/36.00) | 0.53 (0.29-0.95) | 0.032 | 0.502 |
|  | **rs17185905** | **TT/TC+CC** | 90/123 (42.30/57.70) | 72/89 (44.70/55.30) | 0.91 (0.59-1.41) | 0.673 | 0.929 | 48/79 (37.80/62.20) | 72/89 (44.70/55.30) | 0.74 (0.45-1.21) | 0.226 | 0.858 | 42/44 (48.80/51.20) | 72/89 (44.70/55.30) | 1.28 (0.72-2.25) | 0.398 | 0.815 |
|  | **rs1007722** | **CC/TC+TT** | 93/120 (43.70/56.30) | 78/83 (48.40/51.60) | 0.77 (0.5-1.18) | 0.231 | 0.782 | 58/69 (45.70/54.30) | 78/83 (48.40/51.60) | 0.81 (0.50-1.32) | 0.404 | 0.862 | 35/51 (40.70/59.30) | 78/83 (48.40/51.60) | 0.71 (0.40-1.26) | 0.236 | 0.664 |
| ***SCN1A*** | **rs1813502** | **AA/AG+GG** | 92/121 (43.20/56.80) | 67/94 (41.60/58.40) | 1.2 (0.77-1.85) | 0.422 | 0.864 | 53/74 (41.70/58.30) | 67/94 (41.60/58.40) | 1.12 (0.68-1.83) | 0.661 | 0.873 | 39/47 (45.30/54.70) | 67/94 (41.60/58.40) | 1.50 (0.84-2.69) | 0.168 | 0.645 |
|  | **rs10497276** | **CC/AC+AA** | 151/62 (70.90/29.10) | 120/41 (74.50/25.50) | 0.73 (0.45-1.2) | 0.21 | 0.782 | 94/33 (74.00/26.00) | 120/41 (74.50/25.50) | 0.85 (0.49-1.48) | 0.560 | 0.866 | 57/29 (66.30/33.70) | 120/41 (74.50/25.50) | 0.51 (0.27-0.96) | 0.037 | 0.502 |
|  | **rs2298771** | **AA/AG+GG** | 124/89 (58.20/41.80) | 74/87 (46.00/54.00) | 1.49 (0.96-2.29) | 0.073 | 0.734 | 76/51 (59.80/40.20) | 74/87 (46.00/54.00) | 1.67 (1.03-2.73) | 0.038 | 0.604 | 48/38 (55.80/44.20) | 74/87 (46.00/54.00) | 1.23 (0.69-2.17) | 0.481 | 0.815 |
|  | **rs10197430** | **TT/TG+GG** | 102/111 (47.90/52.10) | 91/70 (56.50/43.50) | 0.79 (0.52-1.22) | 0.297 | 0.813 | 53/74 (41.70/58.30) | 91/70 (56.50/43.50) | 0.61 (0.37-0.99) | 0.044 | 0.604 | 49/37 (57.00/43.00) | 91/70 (56.50/43.50) | 1.32 (0.74-2.36) | 0.342 | 0.797 |
|  | **rs6432860** | **GG/AG+AA** | 122/91 (57.30/42.70) | 73/88 (45.30/54.70) | 1.46 (0.95-2.25) | 0.085 | 0.734 | 75/52 (59.10/40.90) | 73/88 (45.30/54.70) | 1.68 (1.03-2.73) | 0.037 | 0.604 | 47/39 (54.70/45.30) | 73/88 (45.30/54.70) | 1.19 (0.67-2.11) | 0.544 | 0.836 |
|  | **rs3812718** | **TT/TC+CC** | 77/136 (36.20/63.80) | 47/114 (29.20/70.80) | 1.16 (0.73-1.85) | 0.532 | 0.869 | 47/80 (37.00/63.00) | 47/114 (29.20/70.80) | 1.25 (0.75-2.10) | 0.395 | 0.862 | 30/56 (34.90/65.10) | 47/114 (29.20/70.80) | 0.98 (0.53-1.80) | 0.937 | 0.976 |
|  | **rs8191987** | **TT/TC+CC** | 151/62 (70.90/29.10) | 124/37 (77.00/23.00) | 0.65 (0.4-1.07) | 0.088 | 0.734 | 94/33 (74.00/26.00) | 124/37 (77.00/23.00) | 0.75 (0.43-1.32) | 0.319 | 0.862 | 57/29 (66.30/33.70) | 124/37 (77.00/23.00) | 0.48 (0.25-0.90) | 0.023 | 0.502 |
|  | **rs10188577** | **TT/TC+CC** | 110/103 (51.60/48.40) | 94/67 (58.40/41.60) | 0.81 (0.53-1.25) | 0.346 | 0.843 | 59/68 (46.50/53.50) | 94/67 (58.40/41.60) | 0.66 (0.41-1.08) | 0.096 | 0.743 | 51/35 (59.30/40.70) | 94/67 (58.40/41.60) | 1.20 (0.67-2.13) | 0.544 | 0.836 |
|  | **rs4667869** | **CC/GC+GG** | 117/96 (54.90/45.10) | 86/75 (53.40/46.60) | 1.09 (0.71-1.67) | 0.708 | 0.929 | 74/53 (58.30/41.70) | 86/75 (53.40/46.60) | 1.22 (0.75-1.98) | 0.432 | 0.862 | 43/43 (50.00/50.00) | 86/75 (53.40/46.60) | 0.90 (0.51-1.58) | 0.705 | 0.900 |
|  | **rs1381105** | **TT/TG+GG** | 67/146 (31.50/68.50) | 60/101 (37.30/62.70) | 0.73 (0.46-1.14) | 0.166 | 0.752 | 45/82 (35.40/64.60) | 60/101 (37.30/62.70) | 0.83 (0.50-1.38) | 0.483 | 0.862 | 22/64 (25.60/74.40) | 60/101 (37.30/62.70) | 0.51 (0.27-0.95) | 0.03 | 0.502 |
|  | **rs6735544** | **AA/AG+GG** | 205/8 (96.20/3.80) | 156/5 (96.90/3.10) | 0.64 (0.2-2.08) | 0.451 | 0.864 | 124/3 (97.60/2.40) | 156/5 (96.90/3.10) | 1.13 (0.26-4.89) | 0.871 | 0.951 | 81/5 (94.20/5.80) | 156/5 (96.90/3.10) | 0.34 (0.08-1.38) | 0.132 | 0.645 |
| ***SLC6A11*** | **rs2272400** | **CC/TC+TT** | 190/23 (89.20/10.80) | 142/19 (88.20/11.80) | 1.11 (0.56-2.2) | 0.775 | 0.929 | 113/14 (89.00/11.00) | 142/19 (88.20/11.80) | 1.05 (0.49-2.27) | 0.903 | 0.951 | - | - | - | - | - |
| ***SYN2*** | **rs6776447** | **AA/AG+GG** | 188/25 (88.30/11.70) | 145/16 (90.10/9.90) | 0.9 (0.45-1.81) | 0.77 | 0.929 | 113/14 (89.00/11.00) | 145/16 (90.10/9.90) | 0.89 (0.41-1.97) | 0.781 | 0.913 | 75/11 (87.20/12.80) | 145/16 (90.10/9.90) | 0.86 (0.35-2.11) | 0.744 | 0.915 |
|  | **rs307588** | **CC/GC+GG** | 132/81 (62.00/38.00) | 108/53 (67.10/32.90) | 0.77 (0.49-1.21) | 0.26 | 0.782 | 78/49 (61.40/38.60) | 108/53 (67.10/32.90) | 0.76 (0.46-1.27) | 0.295 | 0.862 | 54/32 (62.80/37.20) | 108/53 (67.10/32.90) | 0.82 (0.45-1.49) | 0.516 | 0.818 |
|  | **rs160208** | **TT/TC+CC** | 174/39 (81.70/18.30) | 135/26 (83.90/16.10) | 0.84 (0.48-1.48) | 0.549 | 0.874 | 104/23 (81.90/18.10) | 135/26 (83.90/16.10) | 0.88 (0.47-1.67) | 0.706 | 0.895 | 70/16 (81.40/18.60) | 135/26 (83.90/16.10) | 0.84 (0.40-1.75) | 0.638 | 0.890 |
|  | **rs1481864** | **AA/AC+CC** | 185/28 (86.90/13.10) | 148/13 (91.90/8.10) | 0.57 (0.27-1.18) | 0.124 | 0.734 | 112/15 (88.20/11.80) | 148/13 (91.90/8.10) | 0.63 (0.28-1.44) | 0.274 | 0.862 | 73/13 (84.90/15.10) | 148/13 (91.90/8.10) | 0.42 (0.16-1.06) | 0.064 | 0.599 |
|  | **rs3773364** | **AA/AG+GG** | 62/151 (29.10/70.90) | 58/103 (36.00/64.00) | 0.8 (0.51-1.27) | 0.348 | 0.843 | 31/96 (24.40/75.60) | 58/103 (36.00/64.00) | 0.63 (0.37-1.08) | 0.093 | 0.743 | 31/55 (36.00/64.00) | 58/103 (36.00/64.00) | 1.18 (0.65-2.13) | 0.592 | 0.860 |
|  | **rs17035945** | **CC/TC+TT** | 167/46 (78.40/21.60) | 128/33 (79.50/20.50) | 0.89 (0.52-1.51) | 0.653 | 0.923 | 99/28 (78.00/22.00) | 128/33 (79.50/20.50) | 0.88 (0.49-1.60) | 0.677 | 0.887 | 68/18 (79.10/20.90) | 128/33 (79.50/20.50) | 0.92 (0.46-1.85) | 0.813 | 0.952 |
|  | **rs794999** | **GG/AG+AA** | 133/80 (62.40/37.60) | 115/46 (71.40/28.60) | 0.65 (0.41-1.03) | 0.062 | 0.734 | 79/48 (62.20/37.80) | 115/46 (71.40/28.60) | 0.65 (0.39-1.09) | 0.100 | 0.743 | 54/32 (62.80/37.20) | 115/46 (71.40/28.60) | 0.69 (0.38-1.25) | 0.222 | 0.649 |
|  | **rs310766** | **GG/AG+AA** | 135/78 (63.40/36.60) | 115/46 (71.40/28.60) | 0.68 (0.43-1.09) | 0.106 | 0.734 | 80/47 (63.00/37.00) | 115/46 (71.40/28.60) | 0.68 (0.41-1.14) | 0.145 | 0.823 | 55/31 (64.00/36.00) | 115/46 (71.40/28.60) | 0.74 (0.41-1.35) | 0.329 | 0.786 |
| ***GABRA6*** | **rs9313892** | **GG/AG+AA** | 181/32 (85.00/15.00) | 132/29 (82.00/18.00) | 1.34 (0.75-2.38) | 0.321 | 0.843 | 109/18 (85.80/14.20) | 132/29 (82.00/18.00) | 1.39 (0.72-2.69) | 0.321 | 0.862 | 72/14 (83.70/16.30) | 132/29 (82.00/18.00) | 1.18 (0.55-2.52) | 0.664 | 0.900 |
|  | **rs3811995** | **GG/AG+AA** | 73/140 (34.30/65.70) | 57/104 (35.40/64.60) | 0.92 (0.59-1.45) | 0.727 | 0.929 | 42/85 (33.10/66.90) | 57/104 (35.40/64.60) | 0.83 (0.50-1.40) | 0.489 | 0.862 | 31/55 (36.00/64.00) | 57/104 (35.40/64.60) | 1.06 (0.58-1.92) | 0.85 | 0.956 |
|  | **rs13188991** | **GG/AG+AA** | 176/37 (82.60/17.40) | 130/31 (80.70/19.30) | 1.29 (0.74-2.23) | 0.364 | 0.843 | 105/22 (82.70/17.30) | 130/31 (80.70/19.30) | 1.3 (0.70-2.42) | 0.410 | 0.862 | 71/15 (82.60/17.40) | 130/31 (80.70/19.30) | 1.33 (0.65-2.73) | 0.427 | 0.815 |
|  | **rs4454083** | **TT/TC+CC** | 128/85 (60.10/39.90) | 93/68 (57.80/42.20) | 1.18 (0.76-1.84) | 0.448 | 0.864 | 80/47 (63.00/37.00) | 93/68 (57.80/42.20) | 1.29 (0.79-2.14) | 0.310 | 0.862 | 48/38 (55.80/44.20) | 93/68 (57.80/42.20) | 1.03 (0.58-1.83) | 0.911 | 0.976 |
|  | **rs13184586** | **CC/GC+GG** | 70/143 (32.90/67.10) | 53/108 (32.90/67.10) | 0.95 (0.6-1.51) | 0.83 | 0.929 | 40/87 (31.50/68.50) | 53/108 (32.90/67.10) | 0.86 (0.51-1.45) | 0.571 | 0.866 | 30/56 (34.90/65.10) | 53/108 (32.90/67.10) | 1.13 (0.62-2.05) | 0.698 | 0.900 |
|  | **rs11949158** | **GG/AG+AA** | 69/144 (32.40/67.60) | 49/112 (30.40/69.60) | 1.14 (0.71-1.8) | 0.591 | 0.886 | 41/86 (32.30/67.70) | 49/112 (30.40/69.60) | 1.14 (0.68-1.92) | 0.626 | 0.866 | 28/58 (32.60/67.40) | 49/112 (30.40/69.60) | 1.26 (0.69-2.33) | 0.455 | 0.815 |
|  | **rs3811991** | **TT/TG+GG** | 54/159 (25.40/74.60) | 47/114 (29.20/70.80) | 0.8 (0.49-1.3) | 0.374 | 0.843 | 34/93 (26.80/73.20) | 47/114 (29.20/70.80) | 0.83 (0.48-1.44) | 0.510 | 0.862 | 20/66 (23.30/76.70) | 47/114 (29.20/70.80) | 0.77 (0.40-1.48) | 0.426 | 0.815 |
|  | **rs3219151** | **CC/TC+TT** | 70/143 (32.90/67.10) | 54/107 (33.50/66.50) | 0.94 (0.59-1.48) | 0.776 | 0.929 | 40/87 (31.50/68.50) | 54/107 (33.50/66.50) | 0.85 (0.51-1.43) | 0.547 | 0.866 | 30/56 (34.90/65.10) | 54/107 (33.50/66.50) | 1.10 (0.61-2.01) | 0.749 | 0.915 |
| ***GABRA1*** | **rs11575999** | **TT/TC+CC** | 70/143 (32.90/67.10) | 61/100 (37.90/62.10) | 0.84 (0.53-1.31) | 0.439 | 0.864 | 47/80 (37.00/63.00) | 61/100 (37.90/62.10) | 0.97 (0.59-1.60) | 0.899 | 0.951 | 23/63 (26.70/73.30) | 61/100 (37.90/62.10) | 0.62 (0.34-1.15) | 0.127 | 0.645 |
|  | **rs11576001** | **AA/AG+GG** | 76/137 (35.70/64.30) | 55/106 (34.20/65.80) | 1.11 (0.71-1.75) | 0.636 | 0.919 | 44/83 (34.60/65.40) | 55/106 (34.20/65.80) | 1.08 (0.65-1.79) | 0.775 | 0.913 | 32/54 (37.20/62.80) | 55/106 (34.20/65.80) | 1.15 (0.64-2.08) | 0.639 | 0.890 |
|  | **rs12658835** | **AA/AG+GG** | 171/42 (80.30/19.70) | 139/22 (86.30/13.70) | 0.62 (0.34-1.11) | 0.101 | 0.734 | 100/27 (78.70/21.30) | 139/22 (86.30/13.70) | 0.56 (0.30-1.07) | 0.079 | 0.743 | 71/15 (82.60/17.40) | 139/22 (86.30/13.70) | 0.77 (0.35-1.66) | 0.504 | 0.815 |
|  | **rs35166395** | **TT/TC+CC** | 108/105 (50.70/49.30) | 86/75 (53.40/46.60) | 0.89 (0.58-1.37) | 0.589 | 0.886 | 68/59 (53.50/46.50) | 86/75 (53.40/46.60) | 0.94 (0.58-1.52) | 0.787 | 0.913 | 40/46 (46.50/53.50) | 86/75 (53.40/46.60) | 0.78 (0.44-1.38) | 0.391 | 0.815 |
|  | **rs7735530** | **GG/AG+AA** | 137/76 (64.30/35.70) | 97/64 (60.20/39.80) | 1.32 (0.84-2.06) | 0.223 | 0.782 | 77/50 (60.60/39.40) | 97/64 (60.20/39.80) | 1.13 (0.69-1.86) | 0.631 | 0.866 | 60/26 (69.80/30.20) | 97/64 (60.20/39.80) | 1.61 (0.88-2.94) | 0.119 | 0.645 |
|  | **rs12188495** | **TT/TC+CC** | 125/88 (58.70/41.30) | 107/54 (66.50/33.50) | 0.73 (0.47-1.14) | 0.166 | 0.752 | 78/49 (61.40/38.60) | 107/54 (66.50/33.50) | 0.79 (0.48-1.32) | 0.371 | 0.862 | 47/39 (54.70/45.30) | 107/54 (66.50/33.50) | 0.62 (0.34-1.11) | 0.108 | 0.645 |
|  | **rs7732641** | **CC/TC+TT** | 136/77 (63.80/36.20) | 98/63 (60.90/39.10) | 1.3 (0.83-2.03) | 0.257 | 0.782 | 77/50 (60.60/39.40) | 98/63 (60.90/39.10) | 1.13 (0.69-1.86) | 0.629 | 0.866 | 59/27 (68.60/31.40) | 98/63 (60.90/39.10) | 1.51 (0.83-2.76) | 0.174 | 0.645 |
|  | **rs1350372** | **AA/AG+GG** | 66/147 (31.00/69.00) | 47/114 (29.20/70.80) | 1.06 (0.66-1.69) | 0.817 | 0.929 | 43/84 (33.90/66.10) | 47/114 (29.20/70.80) | 1.19 (0.71-2.01) | 0.503 | 0.862 | 23/63 (26.70/73.30) | 47/114 (29.20/70.80) | 0.84 (0.44-1.59) | 0.588 | 0.860 |
|  | **rs1157122** | **TT/TC+CC** | 138/75 (64.80/35.20) | 98/63 (60.90/39.10) | 1.33 (0.85-2.08) | 0.215 | 0.782 | 79/48 (62.20/37.80) | 98/63 (60.90/39.10) | 1.18 (0.72-1.95) | 0.510 | 0.862 | 59/27 (68.60/31.40) | 98/63 (60.90/39.10) | 1.51 (0.83-2.76) | 0.174 | 0.645 |
|  | **rs2279020** | **AA/AG+GG** | 70/143 (32.90/67.10) | 58/103 (36.00/64.00) | 0.93 (0.59-1.47) | 0.754 | 0.929 | 41/86 (32.30/67.70) | 58/103 (36.00/64.00) | 0.87 (0.52-1.44) | 0.580 | 0.866 | 29/57 (33.70/66.30) | 58/103 (36.00/64.00) | 1.02 (0.55-1.86) | 0.96 | 0.976 |
|  | **rs2290732** | **GG/AG+AA** | 70/143 (32.90/67.10) | 58/103 (36.00/64.00) | 0.92 (0.58-1.45) | 0.708 | 0.929 | 43/84 (33.90/66.10) | 58/103 (36.00/64.00) | 0.91 (0.55-1.51) | 0.707 | 0.895 | 27/59 (31.40/68.60) | 58/103 (36.00/64.00) | 0.93 (0.51-1.72) | 0.825 | 0.952 |
|  | **rs998754** | **GG/TG+TT** | 65/148 (30.50/69.50) | 58/103 (36.00/64.00) | 0.8 (0.51-1.27) | 0.347 | 0.843 | 41/86 (32.30/67.70) | 58/103 (36.00/64.00) | 0.84 (0.50-1.40) | 0.496 | 0.862 | 24/62 (27.90/72.10) | 58/103 (36.00/64.00) | 0.75 (0.40-1.40) | 0.364 | 0.815 |
| ***GABRG2*** | **rs3219203** | **CC/TC+TT** | 203/10 (95.30/4.70) | 157/4 (97.50/2.50) | 0.67 (0.2-2.25) | 0.508 | 0.864 | 122/5 (96.10/3.90) | 157/4 (97.50/2.50) | 0.79 (0.20-3.10) | 0.736 | 0.902 | 81/5 (94.20/5.80) | 157/4 (97.50/2.50) | 0.61 (0.15-2.51) | 0.492 | 0.815 |
|  | **rs17060039** | **TT/TC+CC** | 145/68 (68.10/31.90) | 107/54 (66.50/33.50) | 1.05 (0.66-1.67) | 0.833 | 0.929 | 88/39 (69.30/30.70) | 107/54 (66.50/33.50) | 1.29 (0.76-2.19) | 0.337 | 0.862 | 57/29 (66.30/33.70) | 107/54 (66.50/33.50) | 0.82 (0.45-1.49) | 0.513 | 0.818 |
|  | **rs209353** | **CC/TC+TT** | 57/156 (26.80/73.20) | 51/110 (31.70/68.30) | 0.77 (0.48-1.23) | 0.276 | 0.782 | 28/99 (22.00/78.00) | 51/110 (31.70/68.30) | 0.57 (0.33-1.00) | 0.047 | 0.604 | 29/57 (33.70/66.30) | 51/110 (31.70/68.30) | 1.01 (0.56-1.85) | 0.963 | 0.976 |
|  | **rs209358** | **TT/TC+CC** | 91/122 (42.70/57.30) | 76/85 (47.20/52.80) | 0.86 (0.56-1.32) | 0.49 | 0.864 | 49/78 (38.60/61.40) | 76/85 (47.20/52.80) | 0.7 (0.43-1.14) | 0.150 | 0.823 | 42/44 (48.80/51.20) | 76/85 (47.20/52.80) | 1.05 (0.60-1.86) | 0.855 | 0.956 |
|  | **rs211037** | **CC/TC+TT** | 132/81 (62.00/38.00) | 95/66 (59.00/41.00) | 1.22 (0.79-1.9) | 0.375 | 0.843 | 80/47 (63.00/37.00) | 95/66 (59.00/41.00) | 1.22 (0.74-2.00) | 0.440 | 0.862 | 52/34 (60.50/39.50) | 95/66 (59.00/41.00) | 1.17 (0.65-2.09) | 0.595 | 0.860 |
|  | **rs211029** | **CC/TC+TT** | 60/153 (28.20/71.80) | 46/115 (28.60/71.40) | 0.99 (0.62-1.6) | 0.976 | 0.993 | 33/94 (26.00/74.00) | 46/115 (28.60/71.40) | 0.86 (0.50-1.49) | 0.598 | 0.866 | 27/59 (31.40/68.60) | 46/115 (28.60/71.40) | 1.24 (0.67-2.31) | 0.498 | 0.815 |
|  | **rs210983** | **CC/GC+GG** | 132/81 (62.00/38.00) | 85/76 (52.80/47.20) | 1.61 (1.04-2.5) | 0.033 | 0.734 | 75/52 (59.10/40.90) | 85/76 (52.80/47.20) | 1.38 (0.85-2.25) | 0.194 | 0.823 | 57/29 (66.30/33.70) | 85/76 (52.80/47.20) | 1.95 (1.07-3.53) | 0.026 | 0.502 |
|  | **rs2205364** | **GG/GC+CC** | 169/44 (79.30/20.70) | 123/38 (76.40/23.60) | 1.16 (0.69-1.94) | 0.574 | 0.886 | 94/33 (74.00/26.00) | 123/38 (76.40/23.60) | 0.86 (0.49-1.51) | 0.607 | 0.866 | 75/11 (87.20/12.80) | 123/38 (76.40/23.60) | 2.21 (1.02-4.81) | 0.037 | 0.502 |
|  | **rs989694** | **CC/TC+TT** | 65/148 (30.50/69.50) | 51/110 (31.70/68.30) | 1.03 (0.65-1.63) | 0.914 | 0.964 | 39/88 (30.70/69.30) | 51/110 (31.70/68.30) | 1.09 (0.64-1.84) | 0.760 | 0.911 | 26/60 (30.20/69.80) | 51/110 (31.70/68.30) | 0.94 (0.51-1.73) | 0.837 | 0.952 |
|  | **rs211014** | **CC/AC+AA** | 103/110 (48.40/51.60) | 66/95 (41.00/59.00) | 1.3 (0.84-2) | 0.239 | 0.782 | 62/65 (48.80/51.20) | 66/95 (41.00/59.00) | 1.28 (0.79-2.09) | 0.320 | 0.862 | 41/45 (47.70/52.30) | 66/95 (41.00/59.00) | 1.37 (0.78-2.43) | 0.277 | 0.721 |
|  | **rs211013** | **GG/AG+AA** | 64/149 (30.00/70.00) | 53/108 (32.90/67.10) | 0.99 (0.63-1.58) | 0.98 | 0.993 | 39/88 (30.70/69.30) | 53/108 (32.90/67.10) | 1.07 (0.63-1.81) | 0.801 | 0.915 | 25/61 (29.10/70.90) | 53/108 (32.90/67.10) | 0.88 (0.48-1.64) | 0.696 | 0.900 |
|  | **rs424740** | **AA/AT+TT** | 56/157 (26.30/73.70) | 50/111 (31.10/68.90) | 0.87 (0.54-1.4) | 0.566 | 0.886 | 33/94 (26.00/74.00) | 50/111 (31.10/68.90) | 0.88 (0.51-1.50) | 0.632 | 0.866 | 23/63 (26.70/73.30) | 50/111 (31.10/68.90) | 0.87 (0.46-1.62) | 0.654 | 0.900 |
| ***ALDH5A1*** | **rs11759284** | **TT/TG+GG** | 100/113 (46.90/53.10) | 67/94 (41.60/58.40) | 1.38 (0.89-2.13) | 0.15 | 0.752 | 57/70 (44.90/55.10) | 67/94 (41.60/58.40) | 1.26 (0.77-2.06) | 0.355 | 0.862 | 43/43 (50.00/50.00) | 67/94 (41.60/58.40) | 1.52 (0.86-2.70) | 0.15 | 0.645 |
|  | **rs4646828** | **CC/TC+TT** | 100/113 (46.90/53.10) | 60/101 (37.30/62.70) | 1.57 (1.01-2.45) | 0.042 | 0.734 | 57/70 (44.90/55.10) | 60/101 (37.30/62.70) | 1.43 (0.88-2.35) | 0.152 | 0.823 | 43/43 (50.00/50.00) | 60/101 (37.30/62.70) | 1.68 (0.94-2.99) | 0.077 | 0.645 |
|  | **rs4646830** | **CC/GC+GG** | 106/107 (49.80/50.20) | 72/89 (44.70/55.30) | 1.29 (0.84-1.99) | 0.246 | 0.782 | 61/66 (48.00/52.00) | 72/89 (44.70/55.30) | 1.21 (0.75-1.97) | 0.437 | 0.862 | 45/41 (52.30/47.70) | 72/89 (44.70/55.30) | 1.49 (0.84-2.63) | 0.17 | 0.645 |
|  | **rs2817213** | **GG/AG+AA** | 100/113 (46.90/53.10) | 75/86 (46.60/53.40) | 1.09 (0.71-1.68) | 0.695 | 0.929 | 59/68 (46.50/53.50) | 75/86 (46.60/53.40) | 1.07 (0.66-1.74) | 0.787 | 0.913 | 41/45 (47.70/52.30) | 75/86 (46.60/53.40) | 1.18 (0.67-2.09) | 0.559 | 0.850 |
|  | **rs2760118** | **GG/AG+AA** | 101/112 (47.40/52.60) | 70/91 (43.50/56.50) | 1.22 (0.79-1.88) | 0.362 | 0.843 | 55/72 (43.30/56.70) | 70/91 (43.50/56.50) | 1.07 (0.65-1.74) | 0.798 | 0.915 | 46/40 (53.50/46.50) | 70/91 (43.50/56.50) | 1.57 (0.89-2.77) | 0.121 | 0.645 |
|  | **rs2252525** | **CC/AC+AA** | 97/116 (45.50/54.50) | 78/83 (48.40/51.60) | 0.97 (0.63-1.49) | 0.872 | 0.942 | 58/69 (45.70/54.30) | 78/83 (48.40/51.60) | 0.95 (0.58-1.54) | 0.828 | 0.939 | 39/47 (45.30/54.70) | 78/83 (48.40/51.60) | 1.02 (0.58-1.80) | 0.956 | 0.976 |
|  | **rs2247845** | **CC/TC+TT** | 120/93 (56.30/43.70) | 95/66 (59.00/41.00) | 0.96 (0.62-1.48) | 0.854 | 0.935 | 65/62 (51.20/48.80) | 95/66 (59.00/41.00) | 0.76 (0.47-1.23) | 0.263 | 0.862 | 55/31 (64.00/36.00) | 95/66 (59.00/41.00) | 1.30 (0.72-2.33) | 0.38 | 0.815 |
|  | **rs2744601** | **GG/GC+CC** | 173/40 (81.20/18.80) | 127/34 (78.90/21.10) | 1.26 (0.73-2.16) | 0.402 | 0.864 | 107/20 (84.30/15.70) | 127/34 (78.90/21.10) | 1.52 (0.80-2.86) | 0.195 | 0.823 | 66/20 (76.70/23.30) | 127/34 (78.90/21.10) | 0.95 (0.48-1.89) | 0.891 | 0.976 |
|  | **rs1054899** | **CC/AC+AA** | 104/109 (48.80/51.20) | 81/80 (50.30/49.70) | 0.88 (0.57-1.36) | 0.567 | 0.886 | 66/61 (52.00/48.00) | 81/80 (50.30/49.70) | 1.03 (0.64-1.67) | 0.902 | 0.951 | 38/48 (44.20/55.80) | 81/80 (50.30/49.70) | 0.71 (0.40-1.25) | 0.232 | 0.664 |
|  | **rs2744602** | **GG/AG+AA** | 114/99 (53.50/46.50) | 86/75 (53.40/46.60) | 0.95 (0.62-1.47) | 0.827 | 0.929 | 75/52 (59.10/40.90) | 86/75 (53.40/46.60) | 1.23 (0.76-2.01) | 0.399 | 0.862 | 39/47 (45.30/54.70) | 86/75 (53.40/46.60) | 0.62 (0.35-1.10) | 0.103 | 0.645 |
| ***EFHC1*** | **rs3761990** | **AA/AG+GG** | 146/67 (68.50/31.50) | 126/35 (78.30/21.70) | 0.66 (0.4-1.08) | 0.098 | 0.734 | 92/35 (72.40/27.60) | 126/35 (78.30/21.70) | 0.78 (0.44-1.37) | 0.387 | 0.862 | 54/32 (62.80/37.20) | 126/35 (78.30/21.70) | 0.46 (0.24-0.86) | 0.016 | 0.502 |
|  | **rs492153** | **GG/GC+CC** | 143/70 (67.10/32.90) | 106/55 (65.80/34.20) | 1.14 (0.72-1.79) | 0.581 | 0.886 | 87/40 (68.50/31.50) | 106/55 (65.80/34.20) | 1.15 (0.69-1.93) | 0.585 | 0.866 | 56/30 (65.10/34.90) | 106/55 (65.80/34.20) | 1.16 (0.64-2.10) | 0.623 | 0.890 |
|  | **rs12206743** | **GG/GC+CC** | 142/71 (66.70/33.30) | 104/57 (64.60/35.40) | 1.17 (0.75-1.84) | 0.495 | 0.864 | 86/41 (67.70/32.30) | 104/57 (64.60/35.40) | 1.17 (0.70-1.95) | 0.541 | 0.866 | 56/30 (65.10/34.90) | 104/57 (64.60/35.40) | 1.20 (0.66-2.17) | 0.543 | 0.836 |
|  | **rs569275** | **AA/AG+GG** | 137/76 (64.30/35.70) | 108/53 (67.10/32.90) | 1.03 (0.65-1.63) | 0.891 | 0.954 | 85/42 (66.90/33.10) | 108/53 (67.10/32.90) | 1.14 (0.68-1.92) | 0.610 | 0.866 | 52/34 (60.50/39.50) | 108/53 (67.10/32.90) | 0.85 (0.47-1.53) | 0.582 | 0.860 |
|  | **rs3804506** | **GG/AG+AA** | 184/29 (86.40/13.60) | 140/21 (87.00/13.00) | 0.94 (0.5-1.77) | 0.856 | 0.935 | 111/16 (87.40/12.60) | 140/21 (87.00/13.00) | 1 (0.49-2.05) | 0.999 | 0.999 | 73/13 (84.90/15.10) | 140/21 (87.00/13.00) | 0.93 (0.42-2.08) | 0.86 | 0.956 |
|  | **rs17851770** | **AA/AC+CC** | 203/10 (95.30/4.70) | 153/8 (95.00/5.00) | 1.13 (0.41-3.13) | 0.81 | 0.929 | 122/5 (96.10/3.90) | 153/8 (95.00/5.00) | 1.54 (0.46-5.18) | 0.484 | 0.862 | - | - | - | - | - |
|  | **rs7757370** | **AA/AC+CC** | 184/29 (86.40/13.60) | 141/20 (87.60/12.40) | 0.89 (0.47-1.69) | 0.727 | 0.929 | 111/16 (87.40/12.60) | 141/20 (87.60/12.40) | 0.95 (0.46-1.96) | 0.883 | 0.951 | 73/13 (84.90/15.10) | 141/20 (87.60/12.40) | 0.88 (0.39-1.99) | 0.768 | 0.931 |
|  | **rs2397092** | **CC/AC+AA** | 117/96 (54.90/45.10) | 82/79 (50.90/49.10) | 1.05 (0.68-1.62) | 0.821 | 0.929 | 73/54 (57.50/42.50) | 82/79 (50.90/49.10) | 1.25 (0.76-2.03) | 0.378 | 0.862 | 44/42 (51.20/48.80) | 82/79 (50.90/49.10) | 0.77 (0.44-1.37) | 0.38 | 0.815 |
| ***STX1A*** | **rs6956879** | **CC/TC+TT** | 89/124 (41.80/58.20) | 72/89 (44.70/55.30) | 0.85 (0.55-1.32) | 0.472 | 0.864 | 46/81 (36.20/63.80) | 72/89 (44.70/55.30) | 0.66 (0.40-1.09) | 0.104 | 0.743 | 43/43 (50.00/50.00) | 72/89 (44.70/55.30) | 1.43 (0.81-2.54) | 0.216 | 0.645 |
|  | **rs867500** | **GG/GC+CC** | 90/123 (42.30/57.70) | 64/97 (39.80/60.20) | 1.25 (0.8-1.94) | 0.325 | 0.843 | 57/70 (44.90/55.10) | 64/97 (39.80/60.20) | 1.39 (0.85-2.27) | 0.194 | 0.823 | 33/53 (38.40/61.60) | 64/97 (39.80/60.20) | 0.87 (0.48-1.56) | 0.636 | 0.890 |
|  | **rs4363087** | **CC/TC+TT** | 68/145 (31.90/68.10) | 43/118 (26.70/73.30) | 1.19 (0.74-1.92) | 0.473 | 0.864 | 37/90 (29.10/70.90) | 43/118 (26.70/73.30) | 1.01 (0.59-1.74) | 0.973 | 0.998 | 31/55 (36.00/64.00) | 43/118 (26.70/73.30) | 1.90 (1.02-3.54) | 0.044 | 0.502 |
|  | **rs10246419** | **AA/AG+GG** | 68/145 (31.90/68.10) | 54/107 (33.50/66.50) | 0.95 (0.6-1.51) | 0.835 | 0.929 | 36/91 (28.30/71.70) | 54/107 (33.50/66.50) | 0.76 (0.45-1.29) | 0.302 | 0.862 | 32/54 (37.20/62.80) | 54/107 (33.50/66.50) | 1.64 (0.89-3.01) | 0.111 | 0.645 |
|  | **rs941298** | **CC/TC+TT** | 78/135 (36.60/63.40) | 58/103 (36.00/64.00) | 1.09 (0.7-1.71) | 0.702 | 0.929 | 49/78 (38.60/61.40) | 58/103 (36.00/64.00) | 1.2 (0.73-1.99) | 0.476 | 0.862 | 29/57 (33.70/66.30) | 58/103 (36.00/64.00) | 0.80 (0.44-1.47) | 0.471 | 0.815 |
|  | **rs28526693** | **TT/TC+CC** | 208/5 (97.70/2.30) | 148/13 (91.90/8.10) | 2.97 (1-8.84) | 0.041 | 0.734 | 123/4 (96.90/3.10) | 148/13 (91.90/8.10) | 2.18 (0.67-7.09) | 0.172 | 0.823 | 85/1 (98.80/1.20) | 148/13 (91.90/8.10) | 5.52 (0.68-44.95) | 0.051 | 0.502 |
| ***STXBP1*** | **rs1573178** | **GG/AG+AA** | 87/126 (40.80/59.20) | 55/106 (34.20/65.80) | 1.44 (0.92-2.25) | 0.112 | 0.734 | 52/75 (40.90/59.10) | 55/106 (34.20/65.80) | 1.38 (0.83-2.27) | 0.211 | 0.858 | 35/51 (40.70/59.30) | 55/106 (34.20/65.80) | 1.51 (0.84-2.71) | 0.172 | 0.645 |
|  | **rs7852204** | **CC/TC+TT** | 92/121 (43.20/56.80) | 59/102 (36.60/63.40) | 1.33 (0.85-2.06) | 0.209 | 0.782 | 56/71 (44.10/55.90) | 59/102 (36.60/63.40) | 1.33 (0.81-2.19) | 0.254 | 0.862 | 36/50 (41.90/58.10) | 59/102 (36.60/63.40) | 1.31 (0.73-2.34) | 0.361 | 0.815 |
|  | **rs6478788** | **GG/AG+AA** | 90/123 (42.30/57.70) | 63/98 (39.10/60.90) | 0.96 (0.61-1.48) | 0.838 | 0.929 | 54/73 (42.50/57.50) | 63/98 (39.10/60.90) | 1 (0.61-1.63) | 0.985 | 0.998 | 36/50 (41.90/58.10) | 63/98 (39.10/60.90) | 0.91 (0.51-1.62) | 0.742 | 0.915 |
|  | **rs4837172** | **CC/AC+AA** | 112/101 (52.60/47.40) | 81/80 (50.30/49.70) | 1.17 (0.76-1.81) | 0.466 | 0.864 | 69/58 (54.30/45.70) | 81/80 (50.30/49.70) | 1.24 (0.76-2.01) | 0.384 | 0.862 | 43/43 (50.00/50.00) | 81/80 (50.30/49.70) | 1.05 (0.60-1.85) | 0.867 | 0.957 |
|  | **rs10819303** | **GG/AG+AA** | 111/102 (52.10/47.90) | 82/79 (50.90/49.10) | 1.08 (0.7-1.66) | 0.732 | 0.929 | 69/58 (54.30/45.70) | 82/79 (50.90/49.10) | 1.15 (0.71-1.87) | 0.572 | 0.866 | 42/44 (48.80/51.20) | 82/79 (50.90/49.10) | 0.97 (0.55-1.71) | 0.922 | 0.976 |
|  | **rs3824523** | **GG/TG+TT** | 132/81 (62.00/38.00) | 84/77 (52.20/47.80) | 1.6 (1.03-2.47) | 0.035 | 0.734 | 81/46 (63.80/36.20) | 84/77 (52.20/47.80) | 1.74 (1.06-2.85) | 0.028 | 0.604 | 51/35 (59.30/40.70) | 84/77 (52.20/47.80) | 1.35 (0.76-2.40) | 0.3 | 0.744 |
|  | **rs2241167** | **AA/AG+GG** | 82/131 (38.50/61.50) | 50/111 (31.10/68.90) | 1.17 (0.74-1.84) | 0.498 | 0.864 | 51/76 (40.20/59.80) | 50/111 (31.10/68.90) | 1.28 (0.77-2.13) | 0.332 | 0.862 | 31/55 (36.00/64.00) | 50/111 (31.10/68.90) | 1.04 (0.57-1.89) | 0.907 | 0.976 |
| ***GRIN1*** | **rs11146020** | **GG/GC+CC** | 173/40 (81.20/18.80) | 126/35 (78.30/21.70) | 1.61 (0.94-2.77) | 0.084 | 0.734 | 110/17 (86.60/13.40) | 126/35 (78.30/21.70) | 2.43 (1.25-4.75) | 0.007 | 0.604 | 63/23 (73.30/26.70) | 126/35 (78.30/21.70) | 0.99 (0.51-1.90) | 0.971 | 0.977 |
|  | **rs2301364** | **TT/TC+CC** | 91/122 (42.70/57.30) | 75/86 (46.60/53.40) | 0.95 (0.62-1.47) | 0.826 | 0.929 | 54/73 (42.50/57.50) | 75/86 (46.60/53.40) | 0.98 (0.60-1.60) | 0.924 | 0.962 | 37/49 (43.00/57.00) | 75/86 (46.60/53.40) | 0.90 (0.51-1.58) | 0.703 | 0.900 |
|  | **rs4880215** | **CC/AC+AA** | 131/82 (61.50/38.50) | 93/68 (57.80/42.20) | 1.17 (0.75-1.81) | 0.49 | 0.864 | 84/43 (66.10/33.90) | 93/68 (57.80/42.20) | 1.39 (0.84-2.30) | 0.195 | 0.823 | 47/39 (54.70/45.30) | 93/68 (57.80/42.20) | 0.90 (0.51-1.59) | 0.707 | 0.900 |
|  | **rs28425205** | **CC/TC+TT** | 133/80 (62.40/37.60) | 91/70 (56.50/43.50) | 1.32 (0.85-2.05) | 0.214 | 0.782 | 84/43 (66.10/33.90) | 91/70 (56.50/43.50) | 1.51 (0.91-2.49) | 0.108 | 0.743 | 49/37 (57.00/43.00) | 91/70 (56.50/43.50) | 1.11 (0.62-1.97) | 0.726 | 0.915 |
|  | **rs28489906** | **AA/AG+GG** | 93/120 (43.70/56.30) | 64/97 (39.80/60.20) | 1.28 (0.82-1.98) | 0.273 | 0.782 | 65/62 (51.20/48.80) | 64/97 (39.80/60.20) | 1.73 (1.06-2.84) | 0.027 | 0.604 | 28/58 (32.60/67.40) | 64/97 (39.80/60.20) | 0.80 (0.44-1.45) | 0.46 | 0.815 |
|  | **rs6293** | **AA/AG+GG** | 129/84 (60.60/39.40) | 99/62 (61.50/38.50) | 0.95 (0.61-1.47) | 0.805 | 0.929 | 82/45 (64.60/35.40) | 99/62 (61.50/38.50) | 1.08 (0.65-1.79) | 0.761 | 0.911 | 47/39 (54.70/45.30) | 99/62 (61.50/38.50) | 0.79 (0.44-1.40) | 0.412 | 0.815 |
|  | **rs1126442** | **GG/AG+AA** | 128/85 (60.10/39.90) | 101/60 (62.70/37.30) | 0.87 (0.56-1.36) | 0.541 | 0.871 | 81/46 (63.80/36.20) | 101/60 (62.70/37.30) | 0.98 (0.59-1.62) | 0.944 | 0.976 | 47/39 (54.70/45.30) | 101/60 (62.70/37.30) | 0.74 (0.42-1.32) | 0.313 | 0.765 |
| ***SYT1*** | **rs10861034** | **GG/AG+AA** | 123/90 (57.70/42.30) | 90/71 (55.90/44.10) | 1.16 (0.75-1.78) | 0.514 | 0.864 | 73/54 (57.50/42.50) | 90/71 (55.90/44.10) | 1.14 (0.70-1.85) | 0.602 | 0.866 | 50/36 (58.10/41.90) | 90/71 (55.90/44.10) | 1.22 (0.69-2.16) | 0.501 | 0.815 |
|  | **rs7959160** | **GG/GC+CC** | 57/156 (26.80/73.20) | 51/110 (31.70/68.30) | 0.84 (0.53-1.36) | 0.485 | 0.864 | 30/97 (23.60/76.40) | 51/110 (31.70/68.30) | 0.71 (0.41-1.22) | 0.215 | 0.858 | 27/59 (31.40/68.60) | 51/110 (31.70/68.30) | 1.11 (0.60-2.05) | 0.737 | 0.915 |
|  | **rs17046049** | **TT/AT+AA** | 167/46 (78.40/21.60) | 131/30 (81.40/18.60) | 0.87 (0.51-1.48) | 0.597 | 0.887 | 100/27 (78.70/21.30) | 131/30 (81.40/18.60) | 0.87 (0.48-1.59) | 0.658 | 0.873 | 67/19 (77.90/22.10) | 131/30 (81.40/18.60) | 0.91 (0.45-1.84) | 0.794 | 0.946 |
|  | **rs4842438** | **CC/AC+AA** | 177/36 (83.10/16.90) | 135/26 (83.90/16.10) | 0.87 (0.49-1.56) | 0.643 | 0.919 | 106/21 (83.50/16.50) | 135/26 (83.90/16.10) | 0.9 (0.47-1.73) | 0.750 | 0.911 | - | - | - | - | - |
|  | **rs2037743** | **AA/AG+GG** | 56/157 (26.30/73.70) | 44/117 (27.30/72.70) | 0.93 (0.57-1.5) | 0.76 | 0.929 | 28/99 (22.00/78.00) | 44/117 (27.30/72.70) | 0.75 (0.43-1.32) | 0.322 | 0.862 | 28/58 (32.60/67.40) | 44/117 (27.30/72.70) | 1.27 (0.69-2.33) | 0.45 | 0.815 |
|  | **rs941133** | **GG/AG+AA** | 176/37 (82.60/17.40) | 133/28 (82.60/17.40) | 1.00 (0.56-1.77) | 0.999 | 0.999 | 103/24 (81.10/18.90) | 133/28 (82.60/17.40) | 0.88 (0.47-1.66) | 0.703 | 0.895 | 73/13 (84.90/15.10) | 133/28 (82.60/17.40) | 1.35 (0.62-2.96) | 0.447 | 0.815 |
| ***GABRAB3*** | **rs2017247** | **GG/AG+AA** | 79/134 (37.10/62.90) | 75/86 (46.60/53.40) | 0.68 (0.44-1.05) | 0.084 | 0.734 | 44/83 (34.60/65.40) | 75/86 (46.60/53.40) | 0.62 (0.38-1.01) | 0.056 | 0.649 | 35/51 (40.70/59.30) | 75/86 (46.60/53.40) | 0.80 (0.45-1.43) | 0.455 | 0.815 |
|  | **rs3751582** | **TT/TC+CC** | 71/142 (33.30/66.70) | 74/87 (46.99/54.00) | 0.56 (0.36-0.87) | 0.009 | 0.529 | 40/87 (31.50/68.50) | 74/87 (46.00/54.00) | 0.54 (0.33-0.90) | 0.016 | 0.604 | 31/55 (36.00/64.00) | 74/87 (46.00/54.00) | 0.62 (0.34-1.10) | 0.102 | 0.645 |
|  | **rs61998700** | **TT/TC+CC** | 75/138 (35.20/64.80) | 58/103 (36.00/64.00) | 1.00 (0.64-1.57) | 0.993 | 0.999 | 40/87 (31.50/68.50) | 58/103 (36.00/64.00) | 0.8 (0.48-1.34) | 0.397 | 0.862 | 35/51 (40.70/59.30) | 58/103 (36.00/64.00) | 1.47 (0.81-2.65) | 0.204 | 0.645 |
|  | **rs61998701** | **TT/TC+CC** | 69/144 (32.40/67.60) | 42/119 (26.10/73.90) | 1.37 (0.85-2.21) | 0.197 | 0.782 | 38/89 (29.90/70.10) | 42/119 (26.10/73.90) | 1.15 (0.67-1.98) | 0.621 | 0.866 | 31/55 (36.00/64.00) | 42/119 (26.10/73.90) | 1.88 (1.01-3.49) | 0.047 | 0.502 |
|  | **rs17560911** | **CC/GC+GG** | 76/137 (35.70/64.30) | 56/105 (34.80/65.20) | 1.09 (0.69-1.71) | 0.715 | 0.929 | 41/86 (32.30/67.70) | 56/105 (34.80/65.20) | 0.89 (0.53-1.49) | 0.655 | 0.873 | 35/51 (40.70/59.30) | 56/105 (34.80/65.20) | 1.54 (0.85-2.79) | 0.157 | 0.645 |
|  | **rs751994** | **CC/TC+TT** | 79/134 (37.10/62.90) | 60/101 (37.30/62.70) | 0.9 (0.58-1.41) | 0.657 | 0.923 | 49/78 (38.60/61.40) | 60/101 (37.30/62.70) | 1 (0.61-1.65) | 0.985 | 0.998 | 30/56 (34.90/65.10) | 60/101 (37.30/62.70) | 0.73 (0.40-1.32) | 0.295 | 0.744 |
|  | **rs768899** | **CC/TC+TT** | 76/137 (35.70/64.30) | 56/105 (34.80/65.20) | 1.07 (0.68-1.68) | 0.775 | 0.929 | 39/88 (30.70/69.30) | 56/105 (34.80/65.20) | 0.81 (0.48-1.37) | 0.435 | 0.862 | 37/49 (43.00/57.00) | 56/105 (34.80/65.20) | 1.65 (0.91-2.97) | 0.098 | 0.645 |
|  | **rs878960** | **AA/AG+GG** | 82/131 (38.50/61.50) | 55/106 (34.20/65.80) | 1.24 (0.79-1.95) | 0.349 | 0.843 | 55/72 (43.30/56.70) | 55/106 (34.20/65.80) | 1.51 (0.92-2.50) | 0.103 | 0.743 | 27/59 (31.40/68.60) | 55/106 (34.20/65.80) | 0.97 (0.53-1.79) | 0.928 | 0.976 |
|  | **rs4906902** | **AA/AG+GG** | 125/88 (58.70/41.30) | 98/63 (60.90/39.10) | 0.85 (0.55-1.32) | 0.462 | 0.864 | 72/55 (56.70/43.30) | 98/63 (60.90/39.10) | 0.8 (0.49-1.31) | 0.380 | 0.862 | 53/33 (61.60/38.40) | 98/63 (60.90/39.10) | 0.94 (0.52-1.69) | 0.837 | 0.952 |
| ***VAMP2*** | **rs2278637** | **TT/TG+GG** | 122/91 (57.30/42.70) | 79/82 (49.10/50.90) | 1.31 (0.85-2.01) | 0.224 | 0.782 | 70/57 (55.10/44.90) | 79/82 (49.10/50.90) | 1.19 (0.74-1.94) | 0.472 | 0.862 | 52/34 (60.50/39.50) | 79/82 (49.10/50.90) | 1.48 (0.84-2.63) | 0.175 | 0.645 |
|  | **rs1150** | **GG/AG+AA** | 123/90 (57.70/42.30) | 81/80 (50.30/49.70) | 1.29 (0.84-1.99) | 0.248 | 0.782 | 71/56 (55.90/44.10) | 81/80 (50.30/49.70) | 1.2 (0.74-1.95) | 0.465 | 0.862 | 52/34 (60.50/39.50) | 81/80 (50.30/49.70) | 1.45 (0.82-2.58) | 0.198 | 0.645 |
|  | **rs8067606** | **GG/AG+AA** | 123/90 (57.70/42.30) | 81/80 (50.30/49.70) | 1.28 (0.83-1.97) | 0.269 | 0.782 | 71/56 (55.90/44.10) | 81/80 (50.30/49.70) | 1.18 (0.73-1.92) | 0.501 | 0.862 | 52/34 (60.50/39.50) | 81/80 (50.30/49.70) | 1.45 (0.82-2.56) | 0.205 | 0.645 |
|  | **rs9899533** | **GG/GC+CC** | 121/92 (56.80/43.20) | 80/81 (49.70/50.30) | 1.27 (0.83-1.96) | 0.273 | 0.782 | 70/57 (55.10/44.90) | 80/81 (49.70/50.30) | 1.19 (0.73-1.93) | 0.488 | 0.862 | 51/35 (59.30/40.70) | 80/81 (49.70/50.30) | 1.44 (0.81-2.55) | 0.208 | 0.645 |
| ***SCN1B*** | **rs8100085** | **TT/AT+AA** | 122/91 (57.30/42.70) | 73/88 (45.30/54.70) | 1.40 (0.91-2.16) | 0.125 | 0.734 | 72/55 (56.70/43.30) | 73/88 (45.30/54.70) | 1.41 (0.86-2.29) | 0.169 | 0.823 | 50/36 (58.10/41.90) | 73/88 (45.30/54.70) | 1.43 (0.81-2.53) | 0.216 | 0.645 |
|  | **rs55742440** | **TT/TC+CC** | 121/92 (56.80/43.20) | 74/87 (46.00/54.00) | 1.36 (0.88-2.09) | 0.167 | 0.752 | 71/56 (55.90/44.10) | 74/87 (46.00/54.00) | 1.34 (0.83-2.19) | 0.233 | 0.862 | 50/36 (58.10/41.90) | 74/87 (46.00/54.00) | 1.44 (0.81-2.54) | 0.212 | 0.645 |
|  | **rs67777826** | **TT/TC+CC** | 157/56 (73.70/26.30) | 105/56 (65.20/34.80) | 1.31 (0.82-2.09) | 0.256 | 0.782 | 92/35 (72.40/27.60) | 105/56 (65.20/34.80) | 1.25 (0.74-2.12) | 0.409 | 0.862 | 65/21 (75.60/24.40) | 105/56 (65.20/34.80) | 1.51 (0.81-2.84) | 0.194 | 0.645 |
|  | **rs58392252** | **AA/AC+CC** | 167/46 (78.40/21.60) | 120/41 (74.50/25.50) | 1.18 (0.71-1.96) | 0.513 | 0.864 | 100/27 (78.70/21.30) | 120/41 (74.50/25.50) | 1.19 (0.67-2.11) | 0.549 | 0.866 | 67/19 (77.90/22.10) | 120/41 (74.50/25.50) | 1.26 (0.65-2.44) | 0.495 | 0.815 |
|  | **rs2278995** | **TT/TC+CC** | 166/47 (77.90/22.10) | 116/45 (72.00/28.00) | 1.25 (0.76-2.06) | 0.372 | 0.843 | 98/29 (77.20/22.80) | 116/45 (72.00/28.00) | 1.19 (0.68-2.09) | 0.544 | 0.866 | 68/18 (79.10/20.90) | 116/45 (72.00/28.00) | 1.46 (0.75-2.83) | 0.263 | 0.712 |
|  | **rs2278996** | **AA/AC+CC** | 164/49 (77.00/23.00) | 108/53 (67.10/32.90) | 1.52 (0.94-2.45) | 0.089 | 0.734 | 97/30 (76.40/23.60) | 108/53 (67.10/32.90) | 1.46 (0.85-2.52) | 0.170 | 0.823 | 67/19 (77.90/22.10) | 108/53 (67.10/32.90) | 1.74 (0.91-3.33) | 0.087 | 0.645 |
| ***SNAP25*** | **rs6039769** | **CC/AC+AA** | 124/89 (58.20/41.80) | 81/80 (50.30/49.70) | 1.37 (0.88-2.11) | 0.159 | 0.752 | 79/48 (62.20/37.80) | 81/80 (50.30/49.70) | 1.57 (0.96-2.58) | 0.072 | 0.743 | 45/41 (52.30/47.70) | 81/80 (50.30/49.70) | 1.08 (0.61-1.92) | 0.782 | 0.940 |
|  | **rs6032826** | **AA/AG+GG** | 146/67 (68.50/31.50) | 99/62 (61.50/38.50) | 1.43 (0.91-2.25) | 0.124 | 0.734 | 88/39 (69.30/30.70) | 99/62 (61.50/38.50) | 1.42 (0.85-2.38) | 0.175 | 0.823 | 58/28 (67.40/32.60) | 99/62 (61.50/38.50) | 1.40 (0.77-2.57) | 0.271 | 0.720 |
|  | **rs363026** | **CC/AC+AA** | 191/22 (89.70/10.30) | 144/17 (89.40/10.60) | 0.80 (0.39-1.63) | 0.534 | 0.869 | 112/15 (88.20/11.80) | 144/17 (89.40/10.60) | 0.66 (0.30-1.45) | 0.303 | 0.862 | 79/7 (91.90/8.10) | 144/17 (89.40/10.60) | 1.14 (0.42-3.11) | 0.803 | 0.950 |
|  | **rs363014** | **AA/AG+GG** | 109/104 (51.20/48.80) | 103/58 (64.00/36.00) | 0.56 (0.36-0.88) | 0.01 | 0.529 | 67/60 (52.80/47.20) | 103/58 (64.00/36.00) | 0.61 (0.37-1.00) | 0.048 | 0.604 | 42/44 (48.80/51.20) | 103/58 (64.00/36.00) | 0.54 (0.30-0.97) | 0.039 | 0.502 |
|  | **rs12626080** | **CC/GC+GG** | 66/147 (31.00/69.00) | 47/114 (29.20/70.80) | 1.04 (0.65-1.66) | 0.874 | 0.942 | 42/85 (33.10/66.90) | 47/114 (29.20/70.80) | 1.16 (0.69-1.95) | 0.581 | 0.866 | 24/62 (27.90/72.10) | 47/114 (29.20/70.80) | 0.88 (0.47-1.65) | 0.687 | 0.900 |
|  | **rs6133845** | **AA/AG+GG** | 156/57 (73.20/26.80) | 116/45 (72.00/28.00) | 0.98 (0.61-1.59) | 0.943 | 0.986 | 91/36 (71.70/28.30) | 116/45 (72.00/28.00) | 0.96 (0.56-1.64) | 0.873 | 0.951 | 65/21 (75.60/24.40) | 116/45 (72.00/28.00) | 1.02 (0.53-1.96) | 0.949 | 0.976 |
|  | **rs362998** | **CC/TC+TT** | 157/56 (73.70/26.30) | 116/45 (72.00/28.00) | 1.01 (0.62-1.63) | 0.979 | 0.993 | 89/38 (70.10/29.90) | 116/45 (72.00/28.00) | 0.87 (0.51-1.49) | 0.621 | 0.866 | 68/18 (79.10/20.90) | 116/45 (72.00/28.00) | 1.31 (0.67-2.55) | 0.432 | 0.815 |
|  | **rs3787283** | **TT/TC+CC** | 73/140 (34.30/65.70) | 75/86 (46.60/53.40) | 0.55 (0.35-0.85) | 0.007 | 0.529 | 46/81 (36.20/63.80) | 75/86 (46.60/53.40) | 0.54 (0.33-0.90) | 0.016 | 0.604 | 27/59 (31.40/68.60) | 75/86 (46.60/53.40) | 0.60 (0.33-1.08) | 0.088 | 0.645 |
|  | **rs3746544** | **AA/AC+CC** | 113/100 (53.10/46.90) | 76/85 (47.20/52.80) | 1.44 (0.93-2.22) | 0.099 | 0.734 | 64/63 (50.40/49.60) | 76/85 (47.20/52.80) | 1.29 (0.79-2.10) | 0.307 | 0.862 | 49/37 (57.00/43.00) | 76/85 (47.20/52.80) | 1.66 (0.94-2.94) | 0.08 | 0.645 |
|  | **rs1051312** | **TT/TC+CC** | 166/47 (77.90/22.10) | 115/46 (71.40/28.60) | 1.52 (0.92-2.48) | 0.099 | 0.734 | 102/25 (80.30/19.70) | 115/46 (71.40/28.60) | 1.82 (1.02-3.25) | 0.039 | 0.604 | 64/22 (74.40/25.60) | 115/46 (71.40/28.60) | 1.07 (0.57-2.03) | 0.83 | 0.952 |
|  | **rs8636** | **CC/TC+TT** | 113/100 (53.10/46.90) | 76/85 (47.20/52.80) | 1.38 (0.90-2.13) | 0.139 | 0.752 | 65/62 (51.20/48.80) | 76/85 (47.20/52.80) | 1.29 (0.79-2.10) | 0.305 | 0.862 | 48/38 (55.80/44.20) | 76/85 (47.20/52.80) | 1.51 (0.85-2.66) | 0.158 | 0.645 |
| ***GRIK1*** | **rs2832397** | **TT/TC+CC** | 187/26 (87.80/12.20) | 141/20 (87.60/12.40) | 1.17 (0.61-2.23) | 0.637 | 0.919 | 111/16 (87.40/12.60) | 141/20 (87.60/12.40) | 1.04 (0.51-2.14) | 0.908 | 0.951 | 76/10 (88.40/11.60) | 141/20 (87.60/12.40) | 1.44 (0.61-3.45) | 0.401 | 0.815 |
|  | **rs363430** | **CC/TC+TT** | 148/65 (69.50/30.50) | 113/48 (70.20/29.80) | 1.01 (0.63-1.61) | 0.973 | 0.993 | 85/42 (66.90/33.10) | 113/48 (70.20/29.80) | 0.91 (0.54-1.53) | 0.712 | 0.895 | 63/23 (73.30/26.70) | 113/48 (70.20/29.80) | 1.15 (0.61-2.15) | 0.672 | 0.900 |
|  | **rs12626456** | **AA/AG+GG** | 178/35 (83.60/16.40) | 142/19 (88.20/11.80) | 0.63 (0.33-1.19) | 0.146 | 0.752 | 106/21 (83.50/16.50) | 142/19 (88.20/11.80) | 0.61 (0.30-1.23) | 0.166 | 0.823 | 72/14 (83.70/16.30) | 142/19 (88.20/11.80) | 0.66 (0.29-1.52) | 0.333 | 0.786 |
|  | **rs363538** | **AA/AC+CC** | 154/59 (72.30/27.70) | 122/39 (75.80/24.20) | 0.84 (0.51-1.38) | 0.489 | 0.864 | 92/35 (72.40/27.60) | 122/39 (75.80/24.20) | 0.81 (0.47-1.41) | 0.463 | 0.862 | 62/24 (72.10/27.90) | 122/39 (75.80/24.20) | 0.79 (0.41-1.51) | 0.473 | 0.815 |
|  | **rs466476** | **TT/TC+CC** | 92/121 (43.20/56.80) | 71/90 (44.10/55.90) | 0.92 (0.59-1.41) | 0.693 | 0.929 | 54/73 (42.50/57.50) | 71/90 (44.10/55.90) | 0.91 (0.56-1.48) | 0.700 | 0.895 | 38/48 (44.20/55.80) | 71/90 (44.10/55.90) | 1.00 (0.57-1.78) | 0.989 | 0.989 |
|  | **rs2832495** | **CC/TC+TT** | 67/146 (31.50/68.50) | 52/109 (32.30/67.70) | 0.85 (0.53-1.35) | 0.482 | 0.864 | 41/86 (32.30/67.70) | 52/109 (32.30/67.70) | 0.91 (0.55-1.53) | 0.736 | 0.902 | 26/60 (30.20/69.80) | 52/109 (32.30/67.70) | 0.85 (0.46-1.56) | 0.591 | 0.860 |
| **Table contains dominant model P-values adjusted for age and gender calculated by logistic regression. OR, odds ratio; CI, confidence intervals, *FDR corrected for multiple comparisons, none of the SNPs remained significant after correction. rs1266787 (*EFHC1*), rs4837175 (*STXBP1*), rs363504 (*GRIK1*) were non-polymorphic therefore P-values could not be calculated** | | | | | | | | | | | | | | | | | |
|  |  |  |  |  |  |  |  |  |  |  |  |  |  |  |  |  |  |

| **Table S3. Association of variants in all epilepsy patients and epilepsy type subgroups** | | | | | | | | | | | | | | | | | |
| --- | --- | --- | --- | --- | --- | --- | --- | --- | --- | --- | --- | --- | --- | --- | --- | --- | --- |
|  |  |  | **Idiopathic epilepsy cases Vs Controls** | | | | | **Symptomatic epilepsy cases Vs Controls** | | | | | **Cryptogenic epilepsy Cases Vs Controls** | | | | |
| **Gene Name** | **SNP id** | **Dominant model** | **case** | **control** | **OR** | **p-value** | **FDR P-value** | **case** | **control** | **OR** | **p-value** | **FDR P-value** | **case** | **control** | **OR** | **p-value** | **FDR P-value** |
| ***CACNA1E*** | **rs34488539** | **CC/TC+TT** | 21/11 (69.40/30.60) | 102/59 (63.40/36.60) | 1.34 (0.55-3.24) | 0.518 | 0.928 | 48/49 (49.50/50.50) | 102/59 (63.40/36.60) | 0.56 (0.33-0.95) | 0.032 | 0.799 | 56/24 (70.00/30.00) | 102/59 (63.40/36.60) | 1.39 (0.75-2.55) | 0.29 | 0.799 |
|  | **rs4652678** | **TT/TC+CC** | 15/21 (41.70/58.30) | 72/89 (44.70/55.30) | 0.89 (0.37-0.79) | 0.788 | 0.956 | 36/61 (37.10/62.90) | 72/89 (44.70/55.30) | 0.72  (0.42-1.22) | 0.224 | 0.799 | 37/43 (46.20/53.80) | 72/89 (44.70/55.30) | 1.09 (0.61-1.93) | 0.781 | 0.933 |
|  | **rs199930** | **CC/TC+TT** | 15/21 (41.70/58.30) | 76/85 (47.20/52.80) | 0.84 (0.35-0.70) | 0.69 | 0.928 | 41/56 (42.30/57.70) | 76/85 (47.20/52.80) | 0.87  (0.51-1.46) | 0.586 | 0.925 | 38/42 (47.50/52.50) | 76/85 (47.20/52.80) | 1.08 (0.61-1.91) | 0.805 | 0.933 |
|  | **rs704326** | **CC/TC+TT** | 22/14 (61.10/38.90) | 89/72 (55.30/44.70) | 1.48 (0.61-0.38) | 0.379 | 0.923 | 64/33 (66.00/34.00) | 89/72 (55.30/44.70) | 1.64  (0.96-2.81) | 0.068 | 0.799 | 44/36 (55.00/45.00) | 89/72 (55.30/44.70) | 1.09 (0.61-1.95) | 0.774 | 0.933 |
|  | **rs2280869** | **TT/TC+CC** | 22/14 (61.10/38.90) | 120/41 (74.50/25.50) | 0.36 (0.14-0.03) | 0.03 | 0.923 | 72/25 (74.20/25.80) | 120/41 (74.50/25.50) | 0.95  (0.53-1.73) | 0.879 | 0.978 | 65/15 (81.20/18.80) | 120/41 (74.50/25.50) | 1.30 (0.64-2.63) | 0.466 | 0.933 |
|  | **rs590412** | **CC/AC+AA** | 10/26 (27.80/72.20) | 52/109 (32.30/67.70) | 0.51 (0.19-0.18) | 0.176 | 0.923 | 34/63 (35.10/64.90) | 52/109 (32.30/67.70) | 1.17  (0.68-2.02) | 0.579 | 0.925 | 36/44 (45.00/55.00) | 52/109 (32.30/67.70) | 1.75 (0.97-3.15) | 0.064 | 0.799 |
|  | **rs685859** | **GG/GC+CC** | 10/26 (27.80/72.20) | 44/117 (27.30/72.70) | 0.62 (0.22-0.34) | 0.342 | 0.923 | 34/63 (35.10/64.90) | 44/117 (27.30/72.70) | 1.41  (0.81-2.46) | 0.229 | 0.799 | 31/49 (38.80/61.20) | 44/117 (27.30/72.70) | 1.65 (0.90-3.03) | 0.105 | 0.799 |
| ***SCN2A*** | **rs6755708** | **TT/TC+CC** | 23/13 (63.90/36.10) | 87/74 (54.00/46.00) | 1.11 (0.47-0.81) | 0.809 | 0.958 | 66/31 (68.00/32.00) | 87/74 (54.00/46.00) | 1.86  (1.08-3.20) | 0.023 | 0.793 | 50/30 (62.50/37.50) | 87/74 (54.00/46.00) | 1.35 (0.75-2.44) | 0.31 | 0.806 |
|  | **rs6718960** | **AA/AG+GG** | 28/8 (77.80/22.20) | 121/40 (75.20/24.80) | 1.30 (0.47-0.61) | 0.612 | 0.928 | 72/25 (74.20/25.80) | 121/40 (75.20/24.80) | 0.95  (0.52-1.73) | 0.864 | 0.976 | 59/21 (73.80/26.20) | 121/40 (75.20/24.80) | 0.92 (0.48-1.76) | 0.803 | 0.933 |
|  | **rs13432006** | **AA/AG+GG** | 16/20 (44.40/55.60) | 79/82 (49.10/50.90) | 0.60 (0.25-0.24) | 0.241 | 0.923 | 45/52 (46.40/53.60) | 79/82 (49.10/50.90) | 0.88  (0.52-1.49) | 0.64 | 0.925 | 37/43 (46.20/53.80) | 79/82 (49.10/50.90) | 0.95 (0.53-1.69) | 0.861 | 0.940 |
|  | **rs353119** | **GG/AG+AA** | 14/22 (38.90/61.10) | 81/80 (50.30/49.70) | 0.80 (0.34-0.60) | 0.602 | 0.928 | 50/47 (51.50/48.50) | 81/80 (50.30/49.70) | 1.05  (0.63-1.77) | 0.842 | 0.959 | 41/39 (51.20/48.80) | 81/80 (50.30/49.70) | 1.00 (0.56-1.77) | 0.991 | 0.991 |
|  | **rs16850331** | **CC/TC+TT** | 21/15 (58.30/41.70) | 98/63 (60.90/39.10) | 0.65 (0.27-0.33) | 0.327 | 0.923 | 60/37 (61.90/38.10) | 98/63 (60.90/39.10) | 0.94  (0.55-1.60) | 0.823 | 0.950 | 39/41 (48.80/51.20) | 98/63 (60.90/39.10) | 0.57 (0.32-1.01) | 0.054 | 0.799 |
|  | **rs2075703** | **AA/AG+GG** | 21/15 (58.30/41.70) | 91/70 (56.50/43.50) | 0.76 (0.32-0.54) | 0.543 | 0.928 | 56/41 (57.70/42.30) | 91/70 (56.50/43.50) | 0.99  (0.59-1.67) | 0.968 | 0.991 | 38/42 (47.50/52.50) | 91/70 (56.50/43.50) | 0.68 (0.38-1.21) | 0.19 | 0.799 |
|  | **rs2075704** | **GG/AG+AA** | 20/16 (55.60/44.40) | 79/82 (49.10/50.90) | 1.47 (0.62-0.38) | 0.379 | 0.923 | 46/51 (47.40/52.60) | 79/82 (49.10/50.90) | 0.97  (0.58-1.63) | 0.91 | 0.985 | 44/36 (55.00/45.00) | 79/82 (49.10/50.90) | 1.28 (0.72-2.27) | 0.4 | 0.875 |
|  | **rs1947114** | **TT/TC+CC** | 21/15 (58.30/41.70) | 99/62 (61.50/38.50) | 0.63 (0.26-0.29) | 0.295 | 0.923 | 59/38 (60.80/39.20) | 99/62 (61.50/38.50) | 0.89  (0.52-1.51) | 0.66 | 0.925 | 38/42 (47.50/52.50) | 99/62 (61.50/38.50) | 0.54 (0.30-0.96) | 0.036 | 0.799 |
|  | **rs935403** | **AA/AG+GG** | 15/21 (41.70/58.30) | 65/96 (40.40/59.60) | 1.12 (0.47-0.80) | 0.796 | 0.958 | 30/67 (30.90/69.10) | 65/96 (40.40/59.60) | 0.70  (0.41-1.21) | 0.204 | 0.799 | 35/45 (43.80/56.20) | 65/96 (40.40/59.60) | 1.23 (0.69-2.20) | 0.49 | 0.933 |
|  | **rs2060199** | **AA/AT+TT** | 8/28 (22.20/77.80) | 41/120 (25.50/74.50) | 1.04 (0.38-0.94) | 0.939 | 0.965 | 27/70 (27.80/72.20) | 37/124 (23.00/77.00) | 1.16  (0.65-2.10) | 0.613 | 0.925 | 24/56 (30.00/70.00) | 41/120 (25.50/74.50) | 1.49 (0.77-2.88) | 0.238 | 0.799 |
|  | **rs3943809** | **AA/AG+GG** | 21/15 (58.30/41.70) | 103/58 (64.00/36.00) | 0.56 (0.23-0.19) | 0.19 | 0.923 | 65/32 (67.00/33.00) | 103/58 (64.00/36.00) | 1.01  (0.58-1.74) | 0.985 | 0.995 | 44/36 (55.00/45.00) | 103/58 (64.00/36.00) | 0.60 (0.33-1.09) | 0.093 | 0.799 |
|  | **rs17185905** | **TT/TC+CC** | 16/20 (44.40/55.60) | 72/89 (44.70/55.30) | 1.09 (0.46-0.85) | 0.851 | 0.959 | 36/61 (37.10/62.90) | 72/89 (44.70/55.30) | 0.74  (0.43-1.25) | 0.256 | 0.846 | 38/42 (47.50/52.50) | 72/89 (44.70/55.30) | 1.11 (0.63-1.98) | 0.713 | 0.933 |
|  | **rs1007722** | **CC/TC+TT** | 13/23 (36.10/63.90) | 78/83 (48.40/51.60) | 0.49 (0.20-0.10) | 0.104 | 0.923 | 46/51 (47.40/52.60) | 78/83 (48.40/51.60) | 0.90  (0.54-1.51) | 0.689 | 0.925 | 34/46 (42.50/57.50) | 78/83 (48.40/51.60) | 0.73 (0.41-1.30) | 0.278 | 0.799 |
| ***SCN1A*** | **rs1813502** | **AA/AG+GG** | 17/19 (47.20/52.80) | 67/94 (41.60/58.40) | 1.67 (0.70-0.24) | 0.245 | 0.923 | 42/55 (43.30/56.70) | 67/94 (41.60/58.40) | 1.11  (0.66-1.87) | 0.696 | 0.925 | 33/47 (41.20/58.80) | 67/94 (41.60/58.40) | 1.36 (0.75-2.48) | 0.309 | 0.806 |
|  | **rs10497276** | **CC/AC+AA** | 33/3 (91.70/8.30) | 120/41 (74.50/25.50) | 1.70 (0.46-0.40) | 0.406 | 0.928 | 65/32 (67.00/33.00) | 120/41 (74.50/25.50) | 0.65  (0.37-1.16) | 0.146 | 0.799 | 53/27 (66.20/33.80) | 120/41 (74.50/25.50) | 0.53 (0.28-1.00) | 0.05 | 0.799 |
|  | **rs2298771** | **AA/AG+GG** | 20/16 (55.60/44.40) | 74/87 (46.00/54.00) | 1.53 (0.65-0.33) | 0.334 | 0.923 | 55/42 (56.70/43.30) | 74/87 (46.00/54.00) | 1.48  (0.88-2.48) | 0.141 | 0.799 | 49/31 (61.20/38.80) | 74/87 (46.00/54.00) | 1.53 (0.86-2.74) | 0.151 | 0.799 |
|  | **rs10197430** | **TT/TG+GG** | 19/17 (52.80/47.20) | 91/70 (56.50/43.50) | 1.04 (0.44-0.93) | 0.926 | 0.959 | 45/52 (46.40/53.60) | 91/70 (56.50/43.50) | 0.72 (0.43-1.21) | 0.21 | 0.799 | 38/42 (47.50/52.50) | 91/70 (56.50/43.50) | 0.87 (0.49-1.56) | 0.643 | 0.933 |
|  | **rs6432860** | **GG/AG+AA** | 21/15 (58.30/41.70) | 73/88 (45.30/54.70) | 1.64 (0.69-0.26) | 0.257 | 0.923 | 53/44 (54.60/45.40) | 73/88 (45.30/54.70) | 1.38 (0.82-2.32) | 0.22 | 0.799 | 48/32 (60.00/40.00) | 73/88 (45.30/54.70) | 1.56 (0.87-2.78) | 0.135 | 0.799 |
|  | **rs3812718** | **TT/TC+CC** | 17/19 (47.20/52.80) | 47/114 (29.20/70.80) | 1.46 (0.60-0.40) | 0.403 | 0.928 | 33/64 (34.00/66.00) | 47/114 (29.20/70.80) | 1.16 (0.66-2.02) | 0.605 | 0.925 | 27/53 (33.80/66.20) | 47/114 (29.20/70.80) | 0.93 (0.50-1.74) | 0.817 | 0.933 |
|  | **rs8191987** | **TT/TC+CC** | 33/3 (91.70/8.30) | 124/37 (77.00/23.00) | 1.68 (0.45-0.42) | 0.419 | 0.928 | 66/31 (68.00/32.00) | 124/37 (77.00/23.00) | 0.61 (0.34-1.09) | 0.096 | 0.799 | 52/28 (65.00/35.00) | 124/37 (77.00/23.00) | 0.45 (0.24-0.87) | 0.016 | 0.799 |
|  | **rs10188577** | **TT/TC+CC** | 20/16 (55.60/44.40) | 94/67 (58.40/41.60) | 1.10 (0.46-0.83) | 0.828 | 0.958 | 44/53 (45.40/54.60) | 94/67 (58.40/41.60) | 0.61 (0.36-1.02) | 0.058 | 0.799 | 46/34 (57.50/42.50) | 94/67 (58.40/41.60) | 1.17 (0.65-2.10) | 0.604 | 0.933 |
|  | **rs4667869** | **CC/GC+GG** | 15/21 (41.70/58.30) | 86/75 (53.40/46.60) | 0.56 (0.24-0.18) | 0.183 | 0.923 | 57/40 (58.80/41.20) | 86/75 (53.40/46.60) | 1.23 (0.73-2.07) | 0.44 | 0.925 | 45/35 (56.20/43.80) | 86/75 (53.40/46.60) | 1.15 (0.65-2.04) | 0.637 | 0.933 |
|  | **rs1381105** | **TT/TG+GG** | 13/23 (36.10/63.90) | 60/101 (37.30/62.70) | 0.63 (0.26-0.30) | 0.303 | 0.923 | 32/65 (33.00/67.00) | 60/101 (37.30/62.70) | 0.77 (0.44-1.32) | 0.339 | 0.925 | 22/58 (27.50/72.50) | 60/101 (37.30/62.70) | 0.57 (0.31-1.07) | 0.076 | 0.799 |
|  | **rs6735544** | **AA/AG+GG** | 35/1 (97.20/2.80) | 156/5 (96.90/3.10) | 0.52 (0.05-0.60) | 0.602 | 0.928 | 93/4 (95.90/4.10) | 156/5 (96.90/3.10) | 0.64 (0.16-2.56) | 0.536 | 0.925 | 77/3 (96.20/3.80) | 156/5 (96.90/3.10) | 0.61 (0.14-2.76) | 0.531 | 0.933 |
| ***SLC6A11*** | **rs2272400** | **CC/TC+TT** | - | - | - | - | - | - | - | - | - | - | 74/6 (92.50/7.50) | 142/19 (88.20/11.80) | 1.72 (0.62-4.78) | 0.282 | 0.799 |
| ***SYN2*** | **rs6776447** | **AA/AG+GG** | 32/4 (88.90/11.10) | 145/16 (90.10/9.90) | 0.85 (0.21-0.83) | 0.827 | 0.958 | 85/12 (87.60/12.40) | 145/16 (90.10/9.90) | 0.83 (0.36-1.88) | 0.654 | 0.925 | 71/9 (88.80/11.20) | 145/16 (90.10/9.90) | 0.89 (0.35-2.24) | 0.808 | 0.933 |
|  | **rs307588** | **CC/GC+GG** | 24/12 (66.70/33.30) | 108/53 (67.10/32.90) | 0.86 (0.36-0.75) | 0.748 | 0.929 | 61/36 (62.90/37.10) | 108/53 (67.10/32.90) | 0.80 (0.47-1.38) | 0.431 | 0.925 | 47/33 (58.80/41.20) | 108/53 (67.10/32.90) | 0.73 (0.40-1.32) | 0.296 | 0.799 |
|  | **rs160208** | **TT/TC+CC** | 31/5 (86.10/13.90) | 135/26 (83.90/16.10) | 0.91 (0.30-0.87) | 0.87 | 0.959 | 80/17 (82.50/17.50) | 135/26 (83.90/16.10) | 0.90 (0.45-1.79) | 0.77 | 0.925 | 63/17 (78.80/21.20) | 135/26 (83.90/16.10) | 0.78 (0.38-1.60) | 0.498 | 0.933 |
|  | **rs1481864** | **AA/AC+CC** | 32/4 (88.90/11.10) | 148/13 (91.90/8.10) | 0.53 (0.12-0.43) | 0.426 | 0.928 | 83/14 (85.60/14.40) | 148/13 (91.90/8.10) | 0.52 (0.23-1.19) | 0.123 | 0.799 | 70/10 (87.50/12.50) | 148/13 (91.90/8.10) | 0.51 (0.20-1.33) | 0.171 | 0.799 |
|  | **rs3773364** | **AA/AG+GG** | 7/29 (19.40/80.60) | 58/103 (36.00/64.00) | 0.66 (0.25-0.40) | 0.398 | 0.928 | 28/69 (28.90/71.10) | 58/103 (36.00/64.00) | 0.78 (0.44-1.36) | 0.375 | 0.925 | 27/53 (33.80/66.20) | 58/103 (36.00/64.00) | 1.03 (0.56-1.88) | 0.929 | 0.974 |
|  | **rs17035945** | **CC/TC+TT** | 28/8 (77.80/22.20) | 128/33 (79.50/20.50) | 0.95 (0.34-0.92) | 0.922 | 0.959 | 75/22 (77.30/22.70) | 128/33 (79.50/20.50) | 0.85 (0.45-1.61) | 0.626 | 0.925 | 64/16 (80.00/20.00) | 128/33 (79.50/20.50) | 0.96 (0.47-1.96) | 0.912 | 0.963 |
|  | **rs794999** | **GG/AG+AA** | 24/12 (66.70/33.30) | 115/46 (71.40/28.60) | 0.77 (0.31-0.56) | 0.564 | 0.928 | 58/39 (59.80/40.20) | 115/46 (71.40/28.60) | 0.59 (0.34-1.02) | 0.059 | 0.799 | 51/29 (63.80/36.20) | 115/46 (71.40/28.60) | 0.74 (0.40-1.36) | 0.326 | 0.813 |
|  | **rs310766** | **GG/AG+AA** | 24/12 (66.70/33.30) | 115/46 (71.40/28.60) | 0.78 (0.32-0.58) | 0.58 | 0.928 | 60/37 (61.90/38.10) | 115/46 (71.40/28.60) | 0.66 (0.38-1.14) | 0.133 | 0.799 | 51/29 (63.80/36.20) | 115/46 (71.40/28.60) | 0.74 (0.40-1.36) | 0.33 | 0.813 |
| ***GABRA6*** | **rs9313892** | **GG/AG+AA** | 29/7 (80.60/19.40) | 132/29 (82.00/18.00) | 0.74 (0.26-0.58) | 0.582 | 0.928 | 84/13 (86.60/13.40) | 132/29 (82.00/18.00) | 1.58 (0.76-3.26) | 0.211 | 0.799 | 68/12 (85.00/15.00) | 132/29 (82.00/18.00) | 1.21 (0.56-2.64) | 0.625 | 0.933 |
|  | **rs3811995** | **GG/AG+AA** | 12/24 (33.30/66.70) | 57/104 (35.400/64.60) | 0.73 (0.29-0.50) | 0.504 | 0.928 | 31/66 (32.00/68.00) | 57/104 (35.40/64.60) | 0.84 (0.48-1.46) | 0.537 | 0.925 | 30/50 (37.50/62.50) | 57/104 (35.40/64.60) | 1.08 (0.59-1.96) | 0.808 | 0.933 |
|  | **rs13188991** | **GG/AG+AA** | 26/10 (72.20/27.80) | 130/31 (80.70/19.30) | 0.84 (0.32-0.72) | 0.723 | 0.929 | 81/16 (83.50/16.50) | 130/31 (80.70/19.30) | 1.39 (0.70-2.75) | 0.336 | 0.925 | 69/11 (86.20/13.80) | 130/31 (80.70/19.30) | 1.57 (0.72-3.39) | 0.246 | 0.799 |
|  | **rs4454083** | **TT/TC+CC** | 22/14 (61.10/38.90) | 93/68 (57.80/42.20) | 1.15 (0.48-0.75) | 0.753 | 0.929 | 56/41 (57.70/42.30) | 93/68 (57.80/42.20) | 1.03 (0.61-1.75) | 0.904 | 0.985 | 50/30 (62.50/37.50) | 93/68 (57.80/42.20) | 1.37 (0.76-2.47) | 0.29 | 0.799 |
|  | **rs13184586** | **CC/GC+GG** | 12/24 (33.30/66.70) | 53/108 (32.90/67.10) | 0.83 (0.33-0.70) | 0.689 | 0.928 | 30/67 (30.90/69.10) | 53/108 (32.90/67.10) | 0.88 (0.50-1.53) | 0.645 | 0.925 | 28/52 (35.00/65.00) | 53/108 (32.90/67.10) | 1.10 (0.60-2.02) | 0.758 | 0.933 |
|  | **rs11949158** | **GG/AG+AA** | 11/25 (30.60/69.40) | 49/112 (30.40/69.60) | 1.21 (0.48-0.69) | 0.693 | 0.928 | 38/59 (39.20/60.80) | 49/112 (30.40/69.60) | 1.54 (0.89-2.66) | 0.119 | 0.799 | 20/60 (25.00/75.00) | 49/112 (30.40/69.60) | 0.80 (0.42-1.52) | 0.494 | 0.933 |
|  | **rs3811991** | **TT/TG+GG** | 11/25 (30.60/69.40) | 47/114 (29.20/70.80) | 0.85 (0.32-0.73) | 0.734 | 0.929 | 20/77 (20.60/79.40) | 47/114 (29.20/70.80) | 0.64 (0.35-1.18) | 0.143 | 0.799 | 23/57 (28.80/71.20) | 47/114 (29.20/70.80) | 0.98 (0.52-1.86) | 0.961 | 0.991 |
|  | **rs3219151** | **CC/TC+TT** | 12/24 (33.30/66.70) | 54/107 (33.50/66.50) | 0.82 (0.33-0.67) | 0.675 | 0.928 | 29/68 (29.90/70.10) | 54/107 (33.50/66.50) | 0.83 (0.48-1.45) | 0.515 | 0.925 | 29/51 (36.20/63.80) | 54/107 (33.50/66.50) | 1.13 (0.62-2.06) | 0.693 | 0.933 |
| ***GABRA1*** | **rs11575999** | **TT/TC+CC** | 11/25 (30.60/69.40) | 61/100 (37.90/62.10) | 0.83 (0.33-0.69) | 0.692 | 0.928 | 30/67 (30.90/69.10) | 61/100 (37.90/62.10) | 0.76 (0.44-1.31) | 0.319 | 0.925 | 29/51 (36.20/63.80) | 61/100 (37.90/62.10) | 0.91 (0.50-1.64) | 0.751 | 0.933 |
|  | **rs11576001** | **AA/AG+GG** | 12/24 (33.30/66.70) | 55/106 (34.20/65.80) | 0.93 (0.38-0.86) | 0.865 | 0.959 | 39/58 (40.20/59.80) | 55/106 (34.20/65.80) | 1.38 (0.81-2.37) | 0.236 | 0.799 | 25/55 (31.20/68.80) | 55/106 (34.20/65.80) | 0.84 (0.46-1.55) | 0.582 | 0.933 |
|  | **rs12658835** | **AA/AG+GG** | 27/9 (75.00/25.00) | 139/22 (86.30/13.70) | 0.52 (0.19-0.22) | 0.219 | 0.923 | 77/20 (79.40/20.60) | 139/22 (86.30/13.70) | 0.62 (0.31-1.23) | 0.173 | 0.799 | 67/13 (83.80/16.20) | 139/22 (86.30/13.70) | 0.70 (0.32-1.56) | 0.385 | 0.855 |
|  | **rs35166395** | **TT/TC+CC** | 17/19 (47.20/52.80) | 86/75 (53.40/46.60) | 0.79 (0.33-0.59) | 0.593 | 0.928 | 47/50 (48.50/51.50) | 86/75 (53.40/46.60) | 0.82 (0.49-1.38) | 0.448 | 0.925 | 44/36 (55.00/45.00) | 86/75 (53.40/46.60) | 0.94 (0.53-1.67) | 0.834 | 0.933 |
|  | **rs7735530** | **GG/AG+AA** | 22/14 (61.10/38.90) | 97/64 (60.20/39.80) | 1.34 (0.55-0.52) | 0.519 | 0.928 | 65/32 (67.00/33.00) | 97/64 (60.20/39.80) | 1.51 (0.87-2.62) | 0.137 | 0.799 | 50/30 (62.50/37.50) | 97/64 (60.20/39.80) | 1.01 (0.56-1.81) | 0.981 | 0.991 |
|  | **rs12188495** | **TT/TC+CC** | 20/16 (55.60/44.40) | 107/54 (66.50/33.50) | 0.69 (0.29-0.41) | 0.409 | 0.928 | 54/43 (55.70/44.30) | 107/54 (66.50/33.50) | 0.65 (0.38-1.11) | 0.113 | 0.799 | 51/29 (63.80/36.20) | 107/54 (66.50/33.50) | 0.81 (0.44-1.47) | 0.483 | 0.933 |
|  | **rs7732641** | **CC/TC+TT** | 23/13 (63.90/36.10) | 98/63 (60.90/39.10) | 1.62 (0.65-0.29) | 0.293 | 0.923 | 65/32 (67.00/33.00) | 98/63 (60.90/39.10) | 1.47 (0.85-2.55) | 0.166 | 0.799 | 48/32 (60.00/40.00) | 98/63 (60.90/39.10) | 0.94 (0.52-1.69) | 0.836 | 0.933 |
|  | **rs1350372** | **AA/AG+GG** | 14/22 (38.90/61.10) | 47/114 (29.20/70.80) | 1.02 (0.41-0.10) | 0.97 | 0.975 | 27/70 (27.80/72.20) | 47/114 (29.20/70.80) | 0.94 (0.53-1.68) | 0.844 | 0.959 | 25/55 (31.20/68.80) | 47/114 (29.20/70.80) | 1.10 (0.59-2.05) | 0.765 | 0.933 |
|  | **rs1157122** | **TT/TC+CC** | 23/13 (63.90/36.10) | 98/63 (60.90/39.10) | 1.62 (0.65-0.29) | 0.293 | 0.923 | 66/31 (68.00/32.00) | 98/63 (60.90/39.10) | 1.53 (0.88-2.65) | 0.13 | 0.799 | 49/31 (61.20/38.80) | 98/63 (60.90/39.10) | 0.97 (0.54-1.74) | 0.91 | 0.963 |
|  | **rs2279020** | **AA/AG+GG** | 12/24 (33.30/66.70) | 58/103 (36.00/64.00) | 1.22 (0.50-0.66) | 0.661 | 0.928 | 36/61 (37.10/62.90) | 58/103 (36.00/64.00) | 1.14 (0.66-1.96) | 0.638 | 0.925 | 22/58 (27.50/72.50) | 58/103 (36.00/64.00) | 0.59 (0.31-1.11) | 0.095 | 0.799 |
|  | **rs2290732** | **GG/AG+AA** | 12/24 (33.30/66.70) | 58/103 (36.00/64.00) | 1.20 (0.49-0.69) | 0.693 | 0.928 | 36/61 (37.10/62.90) | 58/103 (36.00/64.00) | 1.10 (0.64-1.88) | 0.743 | 0.925 | 22/58 (27.50/72.50) | 58/103 (36.00/64.00) | 0.63 (0.34-1.18) | 0.146 | 0.799 |
|  | **rs998754** | **GG/TG+TT** | 12/24 (33.30/66.70) | 58/103 (36.00/64.00) | 1.07 (0.44-0.88) | 0.878 | 0.959 | 34/63 (35.10/64.90) | 58/103 (36.00/64.00) | 1.00 (0.58-1.72) | 0.992 | 0.995 | 19/61 (23.80/76.20) | 58/103 (36.00/64.00) | 0.49 (0.26-0.94) | 0.028 | 0.799 |
| ***GABRG2*** | **rs3219203** | **CC/TC+TT** | 33/3 (91.70/8.30) | 157/4 (97.50/2.50) | 0.31 (0.06-0.19) | 0.193 | 0.923 | 94/3 (96.90/3.10) | 157/4 (97.50/2.50) | 0.97 (0.21-4.51) | 0.969 | 0.991 | 76/4 (95.00/5.00) | 157/4 (97.50/2.50) | 0.75 (0.17-3.34) | 0.706 | 0.933 |
|  | **rs17060039** | **TT/TC+CC** | 23/13 (63.90/36.10) | 107/54 (66.50/33.50) | 1.31 (0.52-0.56) | 0.563 | 0.928 | 67/30 (69.10/30.90) | 107/54 (66.50/33.50) | 1.09 (0.63-1.91) | 0.75 | 0.925 | 55/25 (68.80/31.20) | 107/54 (66.50/33.50) | 1.15 (0.62-2.13) | 0.657 | 0.933 |
|  | **rs209353** | **CC/TC+TT** | 5/31 (13.90/86.10) | 51/110 (31.70/68.30) | 0.25 (0.07-0.01) | 0.014 | 0.923 | 29/68 (29.90/70.10) | 51/110 (31.70/68.30) | 0.89 (0.51-1.55) | 0.673 | 0.925 | 23/57 (28.80/71.20) | 51/110 (31.70/68.30) | 0.78 (0.42-1.45) | 0.426 | 0.906 |
|  | **rs209358** | **TT/TC+CC** | 11/25 (30.60/69.40) | 76/85 (47.20/52.80) | 0.54 (0.22-0.17) | 0.172 | 0.923 | 44/53 (45.40/54.60) | 76/85 (47.20/52.80) | 0.92 (0.55-1.54) | 0.741 | 0.925 | 36/44 (45.00/55.00) | 76/85 (47.20/52.80) | 0.85 (0.48-1.52) | 0.591 | 0.933 |
|  | **rs211037** | **CC/TC+TT** | 24/12 (66.70/33.30) | 95/66 (59.00/41.00) | 1.87 (0.74-0.17) | 0.174 | 0.923 | 60/37 (61.90/38.10) | 95/66 (59.00/41.00) | 1.15 (0.68-1.96) | 0.599 | 0.925 | 48/32 (60.00/40.00) | 95/66 (59.00/41.00) | 1.11 (0.62-2.00) | 0.722 | 0.933 |
|  | **rs211029** | **CC/TC+TT** | 11/25 (30.60/69.40) | 46/115 (28.60/71.40) | 1.12 (0.44-0.82) | 0.818 | 0.958 | 26/71 (26.80/73.20) | 46/115 (28.60/71.40) | 0.97 (0.54-1.73) | 0.913 | 0.985 | 23/57 (28.80/71.20) | 46/115 (28.60/71.40) | 0.91 (0.48-1.73) | 0.785 | 0.933 |
|  | **rs210983** | **CC/GC+GG** | 24/12 (66.70/33.30) | 85/76 (52.80/47.20) | 1.76 (0.72-0.20) | 0.206 | 0.923 | 61/36 (62.90/37.10) | 85/76 (52.80/47.20) | 1.63 (0.96-2.79) | 0.069 | 0.799 | 47/33 (58.80/41.20) | 85/76 (52.80/47.20) | 1.38 (0.77-2.47) | 0.28 | 0.799 |
|  | **rs2205364** | **GG/GC+CC** | 27/9 (75.00/25.00) | 123/38 (76.40/23.60) | 1.22 (0.45-0.70) | 0.696 | 0.928 | 78/19 (80.40/19.60) | 123/38 (76.40/23.60) | 1.26 (0.67-2.38) | 0.467 | 0.925 | 64/16 (80.00/20.00) | 123/38 (76.40/23.60) | 1.15 (0.57-2.31) | 0.699 | 0.933 |
|  | **rs989694** | **CC/TC+TT** | 9/27 (25.00/75.00) | 51/110 (31.70/68.30) | 0.80 (0.31-0.65) | 0.652 | 0.928 | 34/63 (35.10/64.90) | 51/110 (31.70/68.30) | 1.24 (0.72-2.15) | 0.439 | 0.925 | 22/58 (27.50/72.50) | 51/110 (31.70/68.30) | 0.85 (0.45-1.60) | 0.614 | 0.933 |
|  | **rs211014** | **CC/AC+AA** | 21/15 (58.30/41.70) | 66/95 (41.00/59.00) | 2.13 (0.89-0.08) | 0.085 | 0.923 | 41/56 (42.30/57.70) | 66/95 (41.00/59.00) | 1.06 (0.63-1.79) | 0.822 | 0.950 | 41/39 (51.20/48.80) | 66/95 (41.00/59.00) | 1.46 (0.82-2.60) | 0.197 | 0.799 |
|  | **rs211013** | **GG/AG+AA** | 8/28 (22.20/77.80) | 53/108 (32.90/67.10) | 0.77 (0.29-0.59) | 0.591 | 0.928 | 34/63 (35.10/64.90) | 53/108 (32.90/67.10) | 1.19 (0.69-2.07) | 0.525 | 0.925 | 22/58 (27.50/72.50) | 53/108 (32.90/67.10) | 0.86 (0.46-1.62) | 0.644 | 0.933 |
|  | **rs424740** | **AA/AT+TT** | 8/28 (22.20/77.80) | 50/111 (31.10/68.90) | 0.80 (0.30-0.64) | 0.641 | 0.928 | 31/66 (32.00/68.00) | 50/111 (31.10/68.90) | 1.10 (0.63-1.93) | 0.728 | 0.925 | 17/63 (21.20/78.80) | 50/111 (31.10/68.90) | 0.63 (0.32-1.24) | 0.175 | 0.799 |
| ***ALDH5A1*** | **rs11759284** | **TT/TG+GG** | 19/17 (52.80/47.20) | 67/94 (41.60/58.40) | 1.57 (0.67-0.30) | 0.303 | 0.923 | 45/52 (46.40/53.60) | 67/94 (41.60/58.40) | 1.27 (0.75-2.14) | 0.366 | 0.925 | 36/44 (45.00/55.00) | 67/94 (41.60/58.40) | 1.37 (0.77-2.46) | 0.287 | 0.799 |
|  | **rs4646828** | **CC/TC+TT** | 19/17 (52.80/47.20) | 60/101 (37.30/62.70) | 1.65 (0.70-0.26) | 0.257 | 0.923 | 45/52 (46.40/53.60) | 60/101 (37.30/62.70) | 1.45 (0.86-2.46) | 0.163 | 0.799 | 36/44 (45.00/55.00) | 60/101 (37.30/62.70) | 1.52 (0.85-2.73) | 0.162 | 0.799 |
|  | **rs4646830** | **CC/GC+GG** | 20/16 (55.60/44.40) | 72/89 (44.70/55.30) | 1.96 (0.82-0.13) | 0.126 | 0.923 | 47/50 (48.50/51.50) | 72/89 (44.70/55.30) | 1.23 (0.73-2.06) | 0.442 | 0.925 | 39/41 (48.80/51.20) | 72/89 (44.70/55.30) | 1.23 (0.69-2.19) | 0.474 | 0.933 |
|  | **rs2817213** | **GG/AG+AA** | 20/16 (55.60/44.40) | 75/86 (46.60/53.40) | 1.93 (0.81-0.13) | 0.135 | 0.923 | 45/52 (46.40/53.60) | 75/86 (46.60/53.40) | 1.04 (0.62-1.75) | 0.877 | 0.978 | 35/45 (43.80/56.20) | 75/86 (46.60/53.40) | 1.00 (0.56-1.79) | 0.987 | 0.991 |
|  | **rs2760118** | **GG/AG+AA** | 18/18 (50.00/50.00) | 70/91 (43.50/56.50) | 1.78 (0.75-0.19) | 0.187 | 0.923 | 45/52 (46.40/53.60) | 70/91 (43.50/56.50) | 1.18 (0.70-1.99) | 0.527 | 0.925 | 38/42 (47.50/52.50) | 70/91 (43.50/56.50) | 1.21 (0.68-2.15) | 0.52 | 0.933 |
|  | **rs2252525** | **CC/AC+AA** | 16/20 (44.40/55.60) | 78/83 (48.40/51.60) | 0.86 (0.37-0.73) | 0.73 | 0.929 | 44/53 (45.40/54.60) | 78/83 (48.40/51.60) | 0.98 (0.58-1.65) | 0.931 | 0.991 | 37/43 (46.20/53.80) | 78/83 (48.40/51.60) | 0.95 (0.54-1.69) | 0.87 | 0.940 |
|  | **rs2247845** | **CC/TC+TT** | 16/20 (44.40/55.60) | 95/66 (59.00/41.00) | 0.54 (0.23-0.15) | 0.155 | 0.923 | 56/41 (57.70/42.30) | 95/66 (59.00/41.00) | 0.99 (0.58-1.67) | 0.965 | 0.991 | 48/32 (60.00/40.00) | 95/66 (59.00/41.00) | 1.05 (0.59-1.88) | 0.872 | 0.940 |
|  | **rs2744601** | **GG/GC+CC** | 32/4 (88.90/11.10) | 127/34 (78.90/21.10) | 3.96 (0.97-0.03) | 0.034 | 0.923 | 80/17 (82.50/17.50) | 127/34 (78.90/21.10) | 1.31 (0.67-2.54) | 0.426 | 0.925 | 61/19 (76.20/23.80) | 127/34 (78.90/21.10) | 0.90 (0.45-1.79) | 0.758 | 0.933 |
|  | **rs1054899** | **CC/AC+AA** | 22/14 (61.10/38.90) | 81/80 (50.30/49.70) | 1.84 (0.76-0.17) | 0.169 | 0.923 | 45/52 (46.40/53.60) | 81/80 (50.30/49.70) | 0.83 (0.50-1.40) | 0.494 | 0.925 | 37/43 (46.20/53.80) | 81/80 (50.30/49.70) | 0.75 (0.42-1.34) | 0.334 | 0.813 |
|  | **rs2744602** | **GG/AG+AA** | 24/12 (66.70/33.30) | 86/75 (53.40/46.60) | 2.18 (0.86-0.09) | 0.089 | 0.923 | 53/44 (54.60/45.40) | 86/75 (53.40/46.60) | 1.00 (0.60-1.68) | 0.995 | 0.995 | 37/43 (46.20/53.80) | 86/75 (53.40/46.60) | 0.67 (0.37-1.19) | 0.166 | 0.799 |
| ***EFHC1*** | **rs3761990** | **AA/AG+GG** | 28/8 (77.80/22.20) | 126/35 (78.30/21.70) | 1.20 (0.40-0.75) | 0.747 | 0.929 | 61/36 (62.90/37.10) | 126/35 (78.30/21.70) | 0.49 (0.27-0.87) | 0.014 | 0.793 | 57/23 (71.20/28.80) | 126/35 (78.30/21.70) | 0.70 (0.36-1.36) | 0.294 | 0.799 |
|  | **rs492153** | **GG/GC+CC** | 25/11 (69.40/30.60) | 106/55 (65.80/34.20) | 0.94 (0.39-0.88) | 0.889 | 0.959 | 65/32 (67.00/33.00) | 106/55 (65.80/34.20) | 1.11 (0.65-1.93) | 0.696 | 0.925 | 53/27 (66.20/33.80) | 106/55 (65.80/34.20) | 1.23 (0.67-2.26) | 0.499 | 0.933 |
|  | **rs12206743** | **GG/GC+CC** | 25/11 (69.40/30.60) | 104/57 (64.60/35.40) | 0.95 (0.39-0.90) | 0.907 | 0.959 | 63/34 (64.90/35.10) | 104/57 (64.60/35.40) | 1.07 (0.63-1.84) | 0.796 | 0.934 | 54/26 (67.50/32.50) | 104/57 (64.60/35.40) | 1.36 (0.74-2.49) | 0.324 | 0.813 |
|  | **rs569275** | **AA/AG+GG** | 26/10 (72.20/27.80) | 108/53 (67.10/32.90) | 1.59 (0.60-0.34) | 0.34 | 0.923 | 55/42 (56.70/43.30) | 108/53 (67.10/32.90) | 0.72 (0.42-1.22) | 0.223 | 0.799 | 56/24 (70.00/30.00) | 108/53 (67.10/32.90) | 1.34 (0.72-2.51) | 0.355 | 0.837 |
|  | **rs3804506** | **GG/AG+AA** | 29/7 (80.60/19.40) | 140/21 (87.00/13.00) | 0.64 (0.22-0.43) | 0.435 | 0.928 | 87/10 (89.70/10.30) | 140/21 (87.00/13.00) | 1.29 (0.57-2.91) | 0.542 | 0.925 | 68/12 (85.00/15.00) | 140/21 (87.00/13.00) | 0.89 (0.39-2.02) | 0.787 | 0.933 |
|  | **rs17851770** | **AA/AC+CC** | - | - | - | - | - | 92/5 (94.80/5.20) | 153/8 (95.00/5.00) | 0.98 (0.30-3.21) | 0.971 | 0.991 | 76/4 (95.00/5.00) | 153/8 (95.00/5.00) | 1.20 (0.31-4.54) | 0.792 | 0.933 |
|  | **rs7757370** | **AA/AC+CC** | 29/7 (80.60/19.40) | 141/20 (87.60/12.40) | 0.61 (0.20-0.38) | 0.381 | 0.923 | 87/10 (89.70/10.30) | 141/20 (87.60/12.40) | 1.22 (0.53-2.77) | 0.638 | 0.925 | 68/12 (85.00/15.00) | 141/20 (87.60/12.40) | 0.85 (0.37-1.94) | 0.701 | 0.933 |
|  | **rs2397092** | **CC/AC+AA** | 21/15 (58.30/41.70) | 82/79 (50.90/49.10) | 1.30 (0.55-0.55) | 0.549 | 0.928 | 50/47 (51.50/48.50) | 82/79 (50.90/49.10) | 0.91 (0.54-1.53) | 0.726 | 0.925 | 46/34 (57.50/42.50) | 82/79 (50.90/49.10) | 1.18 (0.66-2.11) | 0.57 | 0.933 |
| ***STX1A*** | **rs6956879** | **CC/TC+TT** | 18/18 (50.00/50.00) | 72/89 (44.70/55.30) | 0.97 (0.41-0.95) | 0.953 | 0.966 | 38/59 (39.20/60.80) | 72/89 (44.70/55.30) | 0.85 (0.50-1.44) | 0.554 | 0.925 | 33/47 (41.20/58.80) | 72/89 (44.70/55.30) | 0.87 (0.48-1.55) | 0.632 | 0.933 |
|  | **rs867500** | **GG/GC+CC** | 16/20 (44.40/55.60) | 64/97 (39.80/60.20) | 1.37 (0.58-0.47) | 0.471 | 0.928 | 39/58 (40.20/59.80) | 64/97 (39.80/60.20) | 1.08 (0.63-1.82) | 0.787 | 0.931 | 35/45 (43.80/56.20) | 64/97 (39.80/60.20) | 1.14 (0.63-2.04) | 0.669 | 0.933 |
|  | **rs4363087** | **CC/TC+TT** | 14/22 (38.90/61.10) | 43/118 (26.70/73.30) | 1.59 (0.62-0.34) | 0.339 | 0.923 | 31/66 (32.00/68.00) | 43/118 (26.70/73.30) | 1.31 (0.74-2.32) | 0.348 | 0.925 | 23/57 (28.80/71.20) | 43/118 (26.70/73.30) | 1.17 (0.61-2.24) | 0.636 | 0.933 |
|  | **rs10246419** | **AA/AG+GG** | 12/24 (33.30/66.70) | 54/107 (33.50/66.50) | 0.95 (0.37-0.91) | 0.913 | 0.959 | 34/63 (35.10/64.90) | 54/107 (33.50/66.50) | 1.16 (0.67-2.00) | 0.602 | 0.925 | 22/58 (27.50/72.50) | 54/107 (33.50/66.50) | 0.87 (0.46-1.65) | 0.677 | 0.933 |
|  | **rs941298** | **CC/TC+TT** | 11/25 (30.60/69.40) | 58/103 (36.00/64.00) | 0.84 (0.34-0.71) | 0.706 | 0.929 | 38/59 (39.20/60.80) | 58/103 (36.00/64.00) | 1.21 (0.71-2.07) | 0.484 | 0.925 | 29/51 (36.20/63.80) | 58/103 (36.00/64.00) | 0.85 (0.46-1.57) | 0.609 | 0.933 |
|  | **rs28526693** | **TT/TC+CC** | 33/3 (91.70/8.30) | 148/13 (91.90/8.10) | 0.49 (0.11-0.36) | 0.366 | 0.923 | 96/1 (99.00/1.00) | 148/13 (91.90/8.10) | 6.74 (0.85-53.16) | 0.021 | 0.793 | 79/1 (98.80/1.20) | 148/13 (91.90/8.10) | 5.35 (0.66-43.51) | 0.055 | 0.799 |
| ***STXBP1*** | **rs1573178** | **GG/AG+AA** | 16/20 (44.40/55.60) | 55/106 (34.20/65.80) | 1.01 (0.41-0.97) | 0.975 | 0.975 | 47/50 (48.50/51.50) | 55/106 (34.20/65.80) | 1.82 (1.07-3.09) | 0.027 | 0.793 | 24/56 (30.00/70.00) | 55/106 (34.20/65.80) | 0.99 (0.54-1.85) | 0.984 | 0.991 |
|  | **rs7852204** | **CC/TC+TT** | 19/17 (52.80/47.20) | 59/102 (36.60/63.40) | 1.28 (0.53-0.58) | 0.58 | 0.928 | 47/50 (48.50/51.50) | 59/102 (36.60/63.40) | 1.57 (0.93-2.66) | 0.092 | 0.799 | 26/54 (32.50/67.50) | 59/102 (36.60/63.40) | 0.92 (0.50-1.70) | 0.796 | 0.933 |
|  | **rs6478788** | **GG/AG+AA** | 12/24 (33.30/66.70) | 63/98 (39.10/60.90) | 0.65 (0.26-0.33) | 0.331 | 0.923 | 40/57 (41.20/58.80) | 63/98 (39.10/60.90) | 0.98 (0.58-1.67) | 0.951 | 0.991 | 38/42 (47.50/52.50) | 63/98 (39.10/60.90) | 1.09 (0.61-1.94) | 0.78 | 0.933 |
|  | **rs4837172** | **CC/AC+AA** | 21/15 (58.30/41.70) | 81/80 (50.30/49.70) | 1.08 (0.45-0.87) | 0.869 | 0.959 | 54/43 (55.70/44.30) | 81/80 (50.30/49.70) | 1.30 (0.77-2.20) | 0.318 | 0.925 | 37/43 (46.20/53.80) | 81/80 (50.30/49.70) | 0.93 (0.53-1.66) | 0.811 | 0.933 |
|  | **rs10819303** | **GG/AG+AA** | 21/15 (58.30/41.70) | 82/79 (50.90/49.10) | 0.93 (0.39-0.88) | 0.879 | 0.959 | 51/46 (52.60/47.40) | 82/79 (50.90/49.10) | 1.09 (0.65-1.83) | 0.746 | 0.925 | 39/41 (48.80/51.20) | 82/79 (50.90/49.10) | 0.99 (0.56-1.76) | 0.985 | 0.991 |
|  | **rs3824523** | **GG/TG+TT** | 25/11 (69.40/30.60) | 84/77 (52.20/47.80) | 1.50 (0.61-0.37) | 0.376 | 0.923 | 60/37 (61.90/38.10) | 84/77 (52.20/47.80) | 1.60 (0.94-2.72) | 0.08 | 0.799 | 47/33 (58.80/41.20) | 84/77 (52.20/47.80) | 1.41 (0.79-2.52) | 0.241 | 0.799 |
|  | **rs2241167** | **AA/AG+GG** | 11/25 (30.60/69.40) | 50/111 (31.10/68.90) | 0.83 (0.33-0.69) | 0.686 | 0.928 | 35/62 (36.10/63.90) | 50/111 (31.10/68.90) | 1.12 (0.65-1.93) | 0.688 | 0.925 | 36/44 (45.00/55.00) | 50/111 (31.10/68.90) | 1.45 (0.80-2.62) | 0.217 | 0.799 |
| ***GRIN1*** | **rs11146020** | **GG/GC+CC** | 31/5 (86.10/13.90) | 126/35 (78.30/21.70) | 2.68 (0.77-0.10) | 0.096 | 0.923 | 79/18 (81.40/18.60) | 126/35 (78.30/21.70) | 1.58 (0.81-3.07) | 0.17 | 0.799 | 63/17 (78.80/21.20) | 126/35 (78.30/21.70) | 1.31 (0.65-2.63) | 0.445 | 0.933 |
|  | **rs2301364** | **TT/TC+CC** | 14/22 (38.90/61.10) | 75/86 (46.60/53.40) | 0.85 (0.36-0.71) | 0.712 | 0.929 | 45/52 (46.40/53.60) | 75/86 (46.60/53.40) | 1.12 (0.66-1.89) | 0.679 | 0.925 | 32/48 (40.00/60.00) | 75/86 (46.60/53.40) | 0.77 (0.43-1.37) | 0.372 | 0.839 |
|  | **rs4880215** | **CC/AC+AA** | 24/12 (66.70/33.30) | 93/68 (57.80/42.20) | 1.41 (0.58-0.44) | 0.441 | 0.928 | 60/37 (61.90/38.10) | 93/68 (57.80/42.20) | 1.11 (0.65-1.88) | 0.709 | 0.925 | 47/33 (58.80/41.20) | 93/68 (57.80/42.20) | 1.20 (0.67-2.15) | 0.55 | 0.933 |
|  | **rs28425205** | **CC/TC+TT** | 24/12 (66.70/33.30) | 91/70 (56.50/43.50) | 1.57 (0.65-0.31) | 0.315 | 0.923 | 61/36 (62.90/37.10) | 91/70 (56.50/43.50) | 1.26 (0.74-2.15) | 0.389 | 0.925 | 48/32 (60.00/40.00) | 91/70 (56.50/43.50) | 1.38 (0.76-2.49) | 0.284 | 0.799 |
|  | **rs28489906** | **AA/AG+GG** | 17/19 (47.20/52.80) | 64/97 (39.80/60.20) | 1.63 (0.68-0.27) | 0.273 | 0.923 | 41/56 (42.30/57.70) | 64/97 (39.80/60.20) | 1.13 (0.67-1.92) | 0.641 | 0.925 | 35/45 (43.80/56.20) | 64/97 (39.80/60.20) | 1.48 (0.82-2.68) | 0.192 | 0.799 |
|  | **rs6293** | **AA/AG+GG** | 24/12 (66.70/33.30) | 99/62 (61.50/38.50) | 1.20 (0.49-0.69) | 0.688 | 0.928 | 59/38 (60.80/39.20) | 99/62 (61.50/38.50) | 0.92 (0.54-1.57) | 0.769 | 0.925 | 46/34 (57.50/42.50) | 99/62 (61.50/38.50) | 0.92 (0.51-1.65) | 0.781 | 0.933 |
|  | **rs1126442** | **GG/AG+AA** | 24/12 (66.70/33.30) | 101/60 (62.70/37.30) | 1.14 (0.47-0.78) | 0.776 | 0.950 | 58/39 (59.80/40.20) | 101/60 (62.70/37.30) | 0.83 (0.49-1.42) | 0.499 | 0.925 | 46/34 (57.50/42.50) | 101/60 (62.70/37.30) | 0.87 (0.48-1.57) | 0.645 | 0.933 |
| ***SYT1*** | **rs10861034** | **GG/AG+AA** | 22/14 (61.10/38.90) | 90/71 (55.90/44.10) | 1.03 (0.43-0.95) | 0.952 | 0.966 | 56/41 (57.70/42.30) | 90/71 (55.90/44.10) | 1.14 (0.67-1.92) | 0.629 | 0.925 | 45/35 (56.20/43.80) | 90/71 (55.90/44.10) | 1.16 (0.65-2.07) | 0.609 | 0.933 |
|  | **rs7959160** | **GG/GC+CC** | 10/26 (27.80/72.20) | 51/110 (31.70/68.30) | 0.72 (0.28-0.50) | 0.499 | 0.928 | 32/65 (33.00/67.00) | 51/110 (31.70/68.30) | 1.16 (0.66-2.01) | 0.61 | 0.925 | 15/65 (18.80/81.20) | 51/110 (31.70/68.30) | 0.53 (0.27-1.07) | 0.068 | 0.799 |
|  | **rs17046049** | **TT/AT+AA** | 25/11 (69.40/30.60) | 131/30 (81.40/18.60) | 0.45 (0.17-0.11) | 0.111 | 0.923 | 78/19 (80.40/19.60) | 131/30 (81.40/18.60) | 0.96 (0.5-1.84) | 0.892 | 0.985 | 64/16 (80.00/20.00) | 131/30 (81.40/18.60) | 1.08 (0.52-2.22) | 0.84 | 0.933 |
|  | **rs4842438** | **CC/AC+AA** | 30/6 (83.30/16.70) | 135/26 (83.90/16.10) | 0.82 (0.28-0.73) | 0.726 | 0.929 | - | - | - | - | - | - | - | - | - | - |
|  | **rs2037743** | **AA/AG+GG** | 9/27 (25.00/75.00) | 44/117 (27.30/72.70) | 0.88 (0.33-0.80) | 0.806 | 0.958 | 22/75 (22.70/77.30) | 44/117 (27.30/72.70) | 0.80 (0.44-1.47) | 0.477 | 0.925 | 25/55 (31.20/68.80) | 44/117 (27.30/72.70) | 1.12 (0.60-2.10) | 0.721 | 0.933 |
|  | **rs941133** | **GG/AG+AA** | - | - | - | - | - | 79/18 (81.40/18.60) | 133/28 (82.60/17.40) | 0.89 (0.45-1.74) | 0.728 | 0.925 | 67/13 (83.80/16.20) | 133/28 (82.60/17.40) | 1.20 (0.55-2.60) | 0.651 | 0.933 |
| ***GABRB3*** | **rs2017247** | **GG/AG+AA** | 10/26 (27.80/72.20) | 75/86 (46.60/53.40) | 0.51 (0.20-0.14) | 0.138 | 0.923 | 45/52 (46.40/53.60) | 75/86 (46.60/53.40) | 0.99 (0.59-1.66) | 0.963 | 0.991 | 24/56 (30.00/70.00) | 75/86 (46.60/53.40) | 0.51 (0.28-0.94) | 0.028 | 0.799 |
|  | **rs3751582** | **TT/TC+CC** | 10/26 (27.80/72.20) | 74/87 (46.00/54.00) | 0.52 (0.21-0.16) | 0.159 | 0.923 | 38/59 (39.20/60.80) | 74/87 (46.00/54.00) | 0.72 (0.42-1.21) | 0.21 | 0.799 | 23/57 (28.80/71.20) | 74/87 (46.00/54.00) | 0.49 (0.27-0.91) | 0.021 | 0.799 |
|  | **rs61998700** | **TT/TC+CC** | 11/25 (30.60/69.40) | 58/103 (36.00/64.00) | 0.65 (0.25-0.37) | 0.367 | 0.923 | 34/63 (35.10/64.90) | 58/103 (36.00/64.00) | 1.02 (0.59-1.75) | 0.945 | 0.991 | 30/50 (37.50/62.50) | 58/103 (36.00/64.00) | 1.10 (0.60-2.00) | 0.759 | 0.933 |
|  | **rs61998701** | **TT/TC+CC** | 12/24 (33.30/66.70) | 42/119 (26.10/73.90) | 0.93 (0.35-0.89) | 0.887 | 0.959 | 30/67 (30.90/69.10) | 42/119 (26.10/73.90) | 1.34 (0.75-2.37) | 0.321 | 0.925 | 27/53 (33.80/66.20) | 42/119 (26.10/73.90) | 1.43 (0.77-2.69) | 0.263 | 0.799 |
|  | **rs17560911** | **CC/GC+GG** | 12/24 (33.30/66.70) | 56/105 (34.80/65.20) | 0.77 (0.31-0.58) | 0.583 | 0.928 | 34/63 (35.10/64.90) | 56/105 (34.80/65.20) | 1.09 (0.63-1.88) | 0.753 | 0.925 | 30/50 (37.50/62.50) | 56/105 (34.80/65.20) | 1.15 (0.63-2.09) | 0.658 | 0.933 |
|  | **rs751994** | **CC/TC+TT** | 13/23 (36.10/63.90) | 60/101 (37.30/62.70) | 0.84 (0.34-0.69) | 0.691 | 0.928 | 40/57 (41.20/58.80) | 60/101 (37.30/62.70) | 1.08 (0.64-1.84) | 0.765 | 0.925 | 26/54 (32.50/67.50) | 60/101 (37.30/62.70) | 0.71 (0.39-1.29) | 0.257 | 0.799 |
|  | **rs768899** | **CC/TC+TT** | 12/24 (33.30/66.70) | 56/105 (34.80/65.20) | 0.75 (0.30-0.55) | 0.548 | 0.928 | 34/63 (35.10/64.90) | 56/105 (34.80/65.20) | 1.08 (0.63-1.86) | 0.779 | 0.929 | 30/50 (37.50/62.50) | 56/105 (34.80/65.20) | 1.10 (0.61-2.00) | 0.75 | 0.933 |
|  | **rs878960** | **AA/AG+GG** | 17/19 (47.20/52.80) | 55/106 (34.20/65.80) | 1.57 (0.66-0.31) | 0.313 | 0.923 | 28/69 (28.90/71.10) | 55/106 (34.20/65.80) | 0.88 (0.50-1.54) | 0.65 | 0.925 | 37/43 (46.20/53.80) | 55/106 (34.20/65.80) | 1.67 (0.92-3.02) | 0.09 | 0.799 |
|  | **rs4906902** | **AA/AG+GG** | 16/20 (44.40/55.60) | 98/63 (60.90/39.10) | 0.44 (0.18-0.06) | 0.063 | 0.923 | 57/40 (58.80/41.20) | 98/63 (60.90/39.10) | 0.90 (0.53-1.53) | 0.702 | 0.925 | 52/28 (65.00/35.00) | 98/63 (60.90/39.10) | 1.08 (0.60-1.96) | 0.792 | 0.933 |
| ***VAMP2*** | **rs2278637** | **TT/TG+GG** | 20/16 (55.60/44.40) | 79/82 (49.10/50.90) | 1.61 (0.68-0.28) | 0.279 | 0.923 | 53/44 (54.60/45.40) | 79/82 (49.10/50.90) | 1.18 (0.70-1.98) | 0.532 | 0.925 | 49/31 (61.20/38.80) | 79/82 (49.10/50.90) | 1.46 (0.81-2.61) | 0.204 | 0.799 |
|  | **rs1150** | **GG/AG+AA** | 20/16 (55.60/44.40) | 81/80 (50.30/49.70) | 1.60 (0.67-0.28) | 0.282 | 0.923 | 53/44 (54.60/45.40) | 81/80 (50.30/49.70) | 1.14 (0.68-1.91) | 0.626 | 0.925 | 50/30 (62.50/37.50) | 81/80 (50.30/49.70) | 1.52 (0.85-2.73) | 0.157 | 0.799 |
|  | **rs8067606** | **GG/AG+AA** | 21/15 (58.30/41.70) | 81/80 (50.30/49.70) | 1.81 (0.75-0.18) | 0.18 | 0.923 | 53/44 (54.60/45.40) | 81/80 (50.30/49.70) | 1.13 (0.68-1.91) | 0.633 | 0.925 | 49/31 (61.20/38.80) | 81/80 (50.30/49.70) | 1.42 (0.79-2.54) | 0.239 | 0.799 |
|  | **rs9899533** | **GG/GC+CC** | 20/16 (55.60/44.40) | 80/81 (49.70/50.30) | 1.69 (0.71-0.23) | 0.231 | 0.923 | 53/44 (54.60/45.40) | 80/81 (49.70/50.30) | 1.17 (0.70-1.97) | 0.551 | 0.925 | 48/32 (60.00/40.00) | 80/81 (49.70/50.30) | 1.40 (0.78-2.49) | 0.256 | 0.799 |
| ***SCN1B*** | **rs8100085** | **TT/AT+AA** | 23/13 (63.90/36.10) | 73/88 (45.30/54.70) | 1.47 (0.62-0.38) | 0.38 | 0.923 | 56/41 (57.70/42.30) | 73/88 (45.30/54.70) | 1.48 (0.88-2.50) | 0.14 | 0.799 | 43/37 (53.80/46.20) | 73/88 (45.30/54.70) | 1.30 (0.73-2.31) | 0.37 | 0.839 |
|  | **rs55742440** | **TT/TC+CC** | 23/13 (63.90/36.10) | 74/87 (46.00/54.00) | 1.51 (0.64-0.35) | 0.347 | 0.923 | 55/42 (56.70/43.30) | 74/87 (46.00/54.00) | 1.40 (0.83-2.36) | 0.206 | 0.799 | 43/37 (53.80/46.20) | 74/87 (46.00/54.00) | 1.30 (0.73-2.31) | 0.37 | 0.839 |
|  | **rs67777826** | **TT/TC+CC** | 27/9 (75.00/25.00) | 105/56 (65.20/34.80) | 1.69 (0.65-0.27) | 0.272 | 0.923 | 69/28 (71.10/28.90) | 105/56 (65.20/34.80) | 1.20 (0.68-2.10) | 0.531 | 0.925 | 61/19 (76.20/23.80) | 105/56 (65.20/34.80) | 1.56 (0.82-2.98) | 0.167 | 0.799 |
|  | **rs58392252** | **AA/AC+CC** | - | - | - | - | - | - | - | - | - | - | 63/17 (78.80/21.20) | 120/41 (74.50/25.50) | 1.24 (0.63-2.44) | 0.536 | 0.933 |
|  | **rs2278995** | **TT/TC+CC** | 29/7 (80.60/19.40) | 116/45 (72.00/28.00) | 1.71 (0.61-0.29) | 0.294 | 0.923 | 74/23 (76.30/23.70) | 116/45 (72.00/28.00) | 1.18 (0.65-2.14) | 0.582 | 0.925 | 63/17 (78.80/21.20) | 116/45 (72.00/28.00) | 1.37 (0.70-2.70) | 0.353 | 0.837 |
|  | **rs2278996** | **AA/AC+CC** | 29/7 (80.60/19.40) | 108/53 (67.10/32.90) | 2.17 (0.78-0.12) | 0.12 | 0.923 | 73/24 (75.30/24.70) | 108/53 (67.10/32.90) | 1.43 (0.80-2.55) | 0.224 | 0.799 | 62/18 (77.50/22.50) | 108/53 (67.10/32.90) | 1.65 (0.86-3.18) | 0.127 | 0.799 |
| ***SNAP25*** | **rs6039769** | **CC/AC+AA** | 22/14 (61.10/38.90) | 81/80 (50.30/49.70) | 1.22 (0.51-0.65) | 0.651 | 0.928 | 57/40 (58.80/41.20) | 81/80 (50.30/49.70) | 1.43 (0.84-2.41) | 0.183 | 0.799 | 45/35 (56.20/43.80) | 81/80 (50.30/49.70) | 1.19 (0.66-2.13) | 0.567 | 0.933 |
|  | **rs6032826** | **AA/AG+GG** | 25/11 (69.40/30.60) | 99/62 (61.50/38.50) | 1.21 (0.48-0.69) | 0.687 | 0.928 | 65/32 (67.00/33.00) | 99/62 (61.50/38.50) | 1.27 (0.74-2.19) | 0.388 | 0.925 | 56/24 (70.00/30.00) | 99/62 (61.50/38.50) | 1.68 (0.90-3.14) | 0.096 | 0.799 |
|  | **rs363026** | **CC/AC+AA** | 32/4 (88.90/11.10) | 144/17 (89.40/10.60) | 0.47 (0.12-0.28) | 0.283 | 0.923 | 85/12 (87.60/12.40) | 144/17 (89.40/10.60) | 0.66 (0.29-1.50) | 0.322 | 0.925 | 74/6 (92.50/7.50) | 144/17 (89.40/10.60) | 1.36 (0.48-3.88) | 0.556 | 0.933 |
|  | **rs363014** | **AA/AG+GG** | 20/16 (55.60/44.40) | 103/58 (64.00/36.00) | 0.74 (0.31-0.50) | 0.498 | 0.928 | 53/44 (54.60/45.40) | 103/58 (64.00/36.00) | 0.69 (0.41-1.16) | 0.161 | 0.799 | 36/44 (45.00/55.00) | 103/58 (64.00/36.00) | 0.44 (0.24-0.79) | 0.006 | 0.799 |
|  | **rs12626080** | **CC/GC+GG** | 8/28 (22.20/77.80) | 47/114 (29.20/70.80) | 0.63 (0.23-0.35) | 0.352 | 0.923 | 31/66 (32.00/68.00) | 47/114 (29.20/70.80) | 1.15 (0.65-2.01) | 0.636 | 0.925 | 27/53 (33.80/66.20) | 47/114 (29.20/70.80) | 1.06 (0.57-1.96) | 0.865 | 0.940 |
|  | **rs6133845** | **AA/AG+GG** | 24/12 (66.70/33.30) | 116/45 (72.00/28.00) | 0.64 (0.25-0.34) | 0.343 | 0.923 | 67/30 (69.10/30.90) | 116/45 (72.00/28.00) | 0.82 (0.47-1.45) | 0.496 | 0.925 | 65/15 (81.20/18.80) | 116/45 (72.00/28.00) | 1.60 (0.80-3.22) | 0.18 | 0.799 |
|  | **rs362998** | **CC/TC+TT** | 24/12 (66.70/33.30) | 116/45 (72.00/28.00) | 0.64 (0.25-0.35) | 0.351 | 0.923 | 67/30 (69.10/30.90) | 116/45 (72.00/28.00) | 0.83 (0.47-1.46) | 0.508 | 0.925 | 66/14 (82.50/17.50) | 116/45 (72.00/28.00) | 1.73 (0.85-3.53) | 0.123 | 0.799 |
|  | **rs3787283** | **TT/TC+CC** | 17/19 (47.20/52.80) | 75/86 (46.60/53.40) | 1.06 (0.45-0.90) | 0.898 | 0.959 | 28/69 (28.90/71.10) | 75/86 (46.60/53.40) | 0.44 (0.25-0.77) | 0.003 | 0.455 | 28/52 (35.00/65.00) | 75/86 (46.60/53.40) | 0.60 (0.33-1.09) | 0.089 | 0.799 |
|  | **rs3746544** | **AA/AC+CC** | 14/22 (38.90/61.10) | 76/85 (47.20/52.80) | 0.81 (0.34-0.64) | 0.64 | 0.928 | 56/41 (57.70/42.30) | 76/85 (47.20/52.80) | 1.65 (0.98-2.79) | 0.058 | 0.799 | 43/37 (53.80/46.20) | 76/85 (47.20/52.80) | 1.54 (0.87-2.75) | 0.14 | 0.799 |
|  | **rs1051312** | **TT/TC+CC** | 27/9 (75.00/25.00) | 115/46 (71.40/28.60) | 1.23 (0.47-0.67) | 0.673 | 0.928 | 75/22 (77.30/22.70) | 115/46 (71.40/28.60) | 1.44 (0.79-2.62) | 0.232 | 0.799 | 64/16 (80.00/20.00) | 115/46 (71.40/28.60) | 1.57 (0.79-3.11) | 0.188 | 0.799 |
|  | **rs8636** | **CC/TC+TT** | 14/22 (38.90/61.10) | 76/85 (47.20/52.80) | 0.79 (0.33-0.59) | 0.593 | 0.928 | 56/41 (57.70/42.30) | 76/85 (47.20/52.80) | 1.59 (0.94-2.67) | 0.082 | 0.799 | 43/37 (53.80/46.20) | 76/85 (47.20/52.80) | 1.51 (0.85-2.70) | 0.159 | 0.799 |
| ***GRIK1*** | **rs2832397** | **TT/TC+CC** | 33/3 (91.70/8.30) | 141/20 (87.60/12.40) | 1.38 (0.36-0.64) | 0.636 | 0.928 | 87/10 (89.70/10.30) | 141/20 (87.60/12.40) | 1.32 (0.58-3.00) | 0.501 | 0.925 | 67/13 (83.80/16.20) | 141/20 (87.60/12.40) | 0.95 (0.42-2.10) | 0.891 | 0.954 |
|  | **rs363430** | **CC/TC+TT** | 22/14 (61.10/38.90) | 113/48 (70.20/29.80) | 0.79 (0.32-0.62) | 0.618 | 0.928 | 66/31 (68.00/32.00) | 113/48 (70.20/29.80) | 0.88 (0.51-1.54) | 0.663 | 0.925 | 60/20 (75.00/25.00) | 113/48 (70.20/29.80) | 1.30 (0.68-2.48) | 0.418 | 0.902 |
|  | **rs12626456** | **AA/AG+GG** | 31/5 (86.10/13.90) | 142/19 (88.20/11.80) | 0.94 (0.25-0.92) | 0.922 | 0.959 | 81/16 (83.50/16.50) | 142/19 (88.20/11.80) | 0.60 (0.28-1.27) | 0.184 | 0.799 | 66/14 (82.50/17.50) | 142/19 (88.20/11.80) | 0.61 (0.27-1.38) | 0.238 | 0.799 |
|  | **rs363538** | **AA/AC+CC** | 27/9 (75.00/25.00) | 122/39 (75.80/24.20) | 0.75 (0.27-0.58) | 0.582 | 0.928 | 72/25 (74.20/25.80) | 122/39 (75.80/24.20) | 0.86 (0.47-1.56) | 0.617 | 0.925 | 55/25 (68.80/31.20) | 122/39 (75.80/24.20) | 0.70 (0.37-1.33) | 0.282 | 0.799 |
|  | **rs466476** | **TT/TC+CC** | 18/18 (50.00/50.00) | 71/90 (44.10/55.90) | 1.28 (0.54-0.58) | 0.576 | 0.928 | 40/57 (41.20/58.80) | 71/90 (44.10/55.90) | 0.89 (0.53-1.50) | 0.659 | 0.925 | 34/46 (42.50/57.50) | 71/90 (44.10/55.90) | 0.94 (0.53-1.67) | 0.831 | 0.933 |
|  | **rs2832495** | **CC/TC+TT** | 13/23 (36.10/63.90) | 52/109 (32.30/67.70) | 1.09 (0.44-0.85) | 0.855 | 0.959 | 28/69 (28.90/71.10) | 52/109 (32.30/67.70) | 0.80 (0.46-1.41) | 0.44 | 0.925 | 26/54 (32.50/67.50) | 52/109 (32.30/67.70) | 0.99 (0.54-1.82) | 0.973 | 0.991 |
| **Table contains dominant model P-values adjusted for age and gender calculated by logistic regression. OR, odds ratio; CI, confidence intervals, *FDR corrected for multiple comparisons, none of the SNPs remained significant after correction. rs1266787 (*EFHC1*), rs4837175 (*STXBP1*), rs363504 (*GRIK1*) were non-polymorphic therefore P-values could not be calculated** | | | | | | | | | | | | | | | | | |
|  |  |  |  |  |  |  |  |  |  |  |  |  |  |  |  |  |  |

**Table S4. Gene-gene interaction results for best models among *SVC* and ion-channel genes in all epilepsy patients and different subgroups.**

| **Model** | **Accuracy** | **Sensitivity** | **Specificity** | **Cross validation consistency (CVC)*** | **OR (95% CI)** | **P-value** | **P-value 1000 permutation** |
| --- | --- | --- | --- | --- | --- | --- | --- |
| **Generalized** |  |  |  |  |  |  |  |
| ***GABRB3*_rs878960, *SCN1A*_rs1813502** | 65.10 | 56.25 | 71.76 | 10/10 | 3.27 (2.01-5.30) | P<0.0001 | 0.490 |

The interaction model was an output of multidimensionality reduction (MDR). * CVC>8/10
